# Supplementary material for: Mll-AF4 Confers Enhanced Self-Renewal and Lymphoid Potential during a Restricted Window in Development
Source: Cell Rep. 2016 Jul 7;16(4):1039–54. doi: 10.1016/j.celrep.2016.06.046 (PMC4967476; doi:10.1016/j.celrep.2016.06.046)
Supplement: Document S1. Figures S1 and S2 and Table S1 [file mmc1.pdf]

Cell Reports, Volume *16*

Supplemental Information

Mll-AF4 Confers Enhanced Self-Renewal and Lymphoid Potential during a Restricted Window in Development

Neil A. Barrett, Camille Malouf, Chrysa Kapeni, Wendi A. Bacon, George Giotopoulos, Sten Eirik W. Jacobsen, Brian J. Huntly, and Katrin Ottersbach

Supplemental Figures:

Figure S1: Mll-AF4 does not cause major changes to embryonic hematopoiesis. Related to Figure 2.

The proportion of distinct hematopoietic cell populations was determined in embryonic tissues of indicated genotypes by flow cytometry. (A) HSPCs in the E11 YS, n=3-10; (B) HSPCs, n=3-6, and (C) B cells in the E11 AGM, n=3-4; (D) HSPCs, n=3-10; (E) B cells, n=3-10 and (F) LMPPs (Lin- B220- CD19- ckit+ CD45+ Sca1+ Flt3+) in the E12 FL, n=2-7; (G) ESLAM (EPCR+ CD45+ CD150+ CD48-), n=5-8; (H) B cells, n=3-9, and (I) LMPPs in the E14 FL, n=3-7. Error bars = SEM. Statistical analysis was performed using the non-parametric Kruskal-Wallis test with Dunn's multiple comparisons, but no significant differences were found.

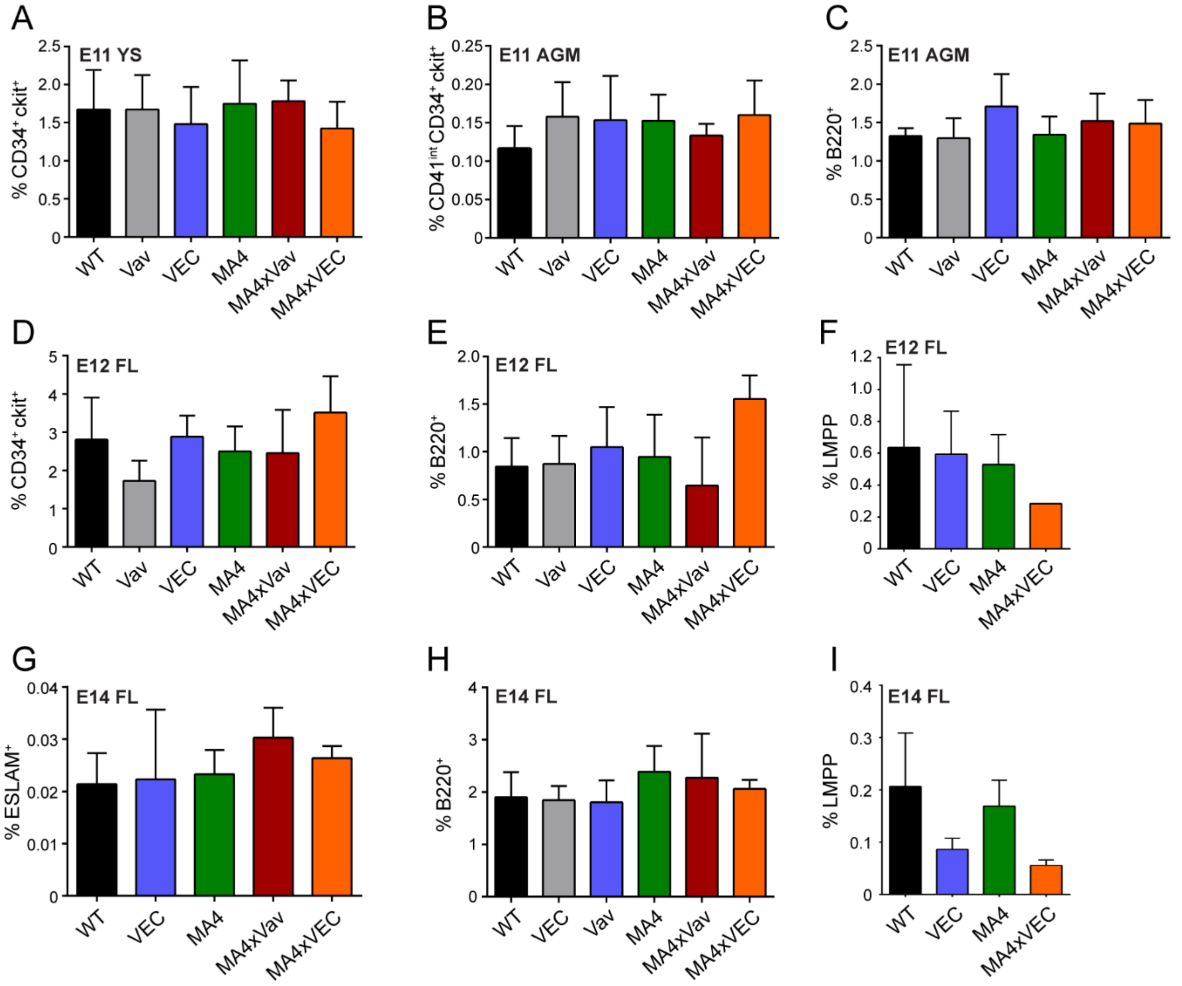

Figure S2: Mll-AF4 recruits SEC and DOT1L. Related to Figure 4.

Effect of inhibitors of BET proteins (IBET) and DOT1L (SGC, EPZ) at indicated concentrations (μM) on B lymphoid colony formation from (A) E14 FL Mll-AF4-expressing cells, n=3-4; (B) E14 FL Cre-only control cells, n=2; (C) adult BM (~1 year old, pre-morbid) Mll-AF4-expressing cells, n=2; (D) adult BM (~1 year old, pre-morbid) Cre-only control cells, n=2. Error bars = SEM. Statistical analysis was performed using a parametric one-way ANOVA with Tukey's multiple comparisons test. **** p<0.0001, *** p<0.001, ** p<0.01.

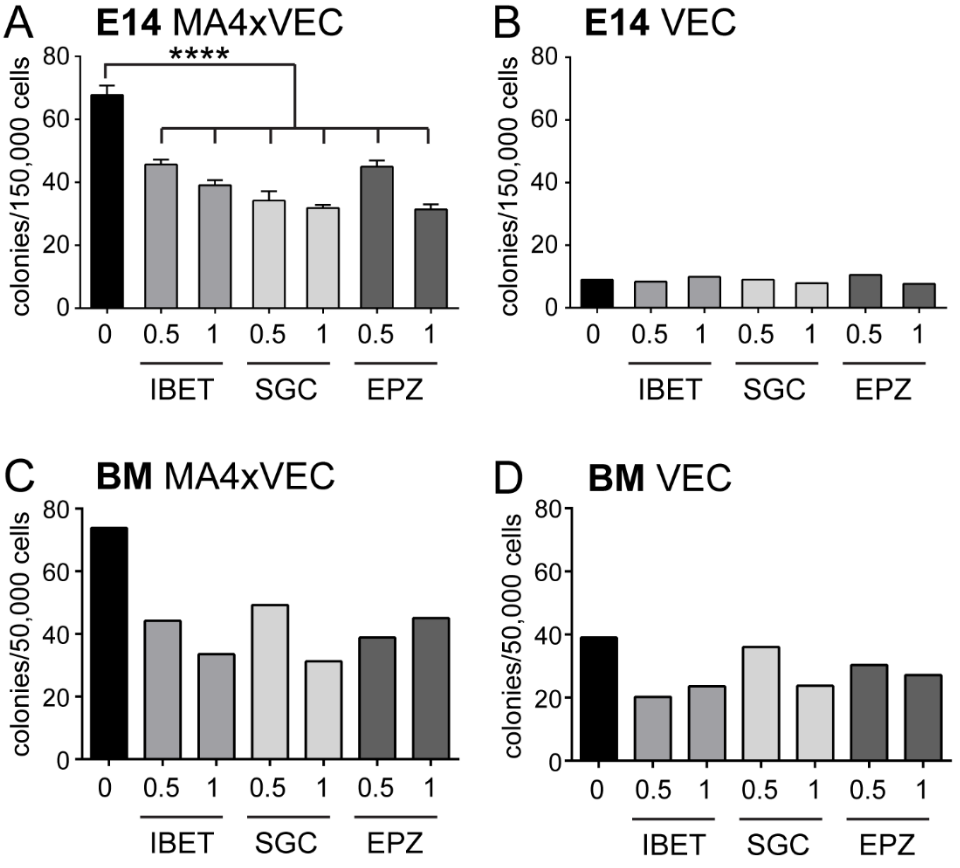

Supplemental Table S1: Disease development in transplant recipients. Related to Figure 5.

	MA4xVEC				VEC		
	No.sick/ total	latency (days)	% donor chimerism	phenotype	No. sick/total	% donor chimerism	phenotype
Primary							
E12 Plac	1/6	186	0	no donor cells<br>expansion of recipient T cells	0/6		
E12 FL	5/7	293-405	95-100	splenomegaly<br>hepatomegaly<br>mesenteric tumor<br>mediastinal disease<br>reduced B cells<br>increased myeloid cells	0/5		
E14 FL	1/14	345	95-100	slight splenomegaly<br>reduced B cells<br>increased myeloid cells<br>increased T cells<br>increased ckit+ cells	1/10	6	expansion of recipient myeloid cells
	2/14	150-186	0-10	slight hepatomegaly<br>expansion of recipient T cells			
Secondary							
E14 FL	1/10	280	95-100	splenomegaly<br>increased myeloid cells<br>increased T cells	1/11	0	splenomegaly<br>slight hepatomegaly<br>reduced recipient leukocytes
