# Supplementary material for: Mll-AF4 Confers Enhanced Self-Renewal and Lymphoid Potential during a Restricted Window in Development
Source: Cell Rep. 2016 Jul 7;16(4):1039–54. doi: 10.1016/j.celrep.2016.06.046 (PMC4967476; doi:10.1016/j.celrep.2016.06.046)
Supplement: Document S2. Article plus Supplemental Information [file mmc2.pdf]

Cell Reports

Article

Mll-AF4 Confers Enhanced Self-Renewal and Lymphoid Potential during a Restricted Window in Development

Graphical Abstract

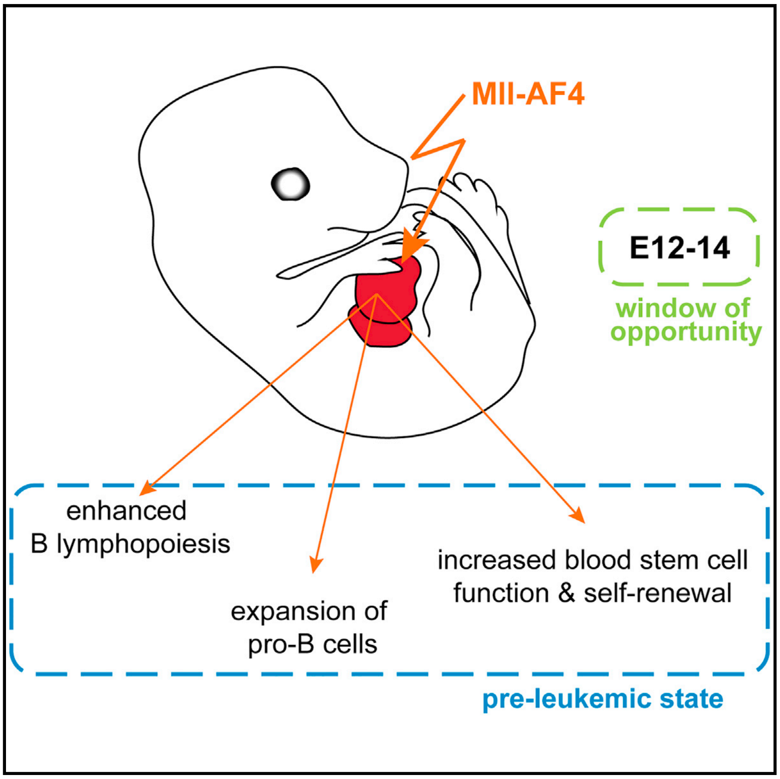

Authors

Neil A. Barrett, Camille Malouf, Chrysa Kapeni, ..., Sten Eirik W. Jacobsen, Brian J. Huntly, Katrin Ottersbach

Correspondence

katrin.ottersbach@ed.ac.uk

In Brief

Barrett et al. describe the changes in prenatal hematopoiesis induced by the Mll-AF4 oncogene in vivo. These include enhanced B lymphoid potential, an expansion of pro-B cells, and increased self-renewal. The authors identify the midgestation lymphoid-primed multipotent progenitor as a potential cell of origin.

Highlights

- Mll-AF4 confers enhanced B cell potential and causes an expansion of pro-B cells
- Mll-AF4 increases self-renewal potential
- Mll-AF4 exerts its effects in a restricted developmental window
- The LMPP is a potential cell of origin for Mll-AF4-associated disease

Barrett et al., 2016, Cell Reports *16*, 1039–1054
July 26, 2016 © 2016 The Author(s).
http://dx.doi.org/10.1016/j.celrep.2016.06.046

Cell Reports
Article

OPEN ACCESS
CellPress

Mll-AF4 Confers Enhanced Self-Renewal and Lymphoid Potential during a Restricted Window in Development

Neil A. Barrett,[1,4] Camille Malouf,[1,2,4] Chrysa Kapeni,[1,2] Wendi A. Bacon,[1] George Giotopoulos,[1] Sten Eirik W. Jacobsen,[3] Brian J. Huntly,[1] and Katrin Ottersbach[1,2,*]
[1]Department of Haematology, Cambridge Institute for Medical Research, Wellcome Trust-Medical Research Council Cambridge Stem Cell Institute, University of Cambridge, Cambridge CB2 0XY, UK
[2]MRC Centre for Regenerative Medicine, University of Edinburgh, Edinburgh EH16 4UU, UK
[3]Haematopoietic Stem Cell Biology Laboratory, MRC Molecular Haematology Unit, Weatherall Institute for Molecular Medicine, University of Oxford, Oxford OX3 9DS, UK
[4]Co-first author
*Correspondence: katrin.ottersbach@ed.ac.uk
http://dx.doi.org/10.1016/j.celrep.2016.06.046

SUMMARY

MLL-AF4+ infant B cell acute lymphoblastic leukemia is characterized by an early onset and dismal survival. It initiates before birth, and very little is known about the early stages of the disease's development. Using a conditional Mll-AF4-expressing mouse model in which fusion expression is targeted to the earliest definitive hematopoietic cells generated in the mouse embryo, we demonstrate that Mll-AF4 imparts enhanced B lymphoid potential and increases repopulation and self-renewal capacity during a putative pre-leukemic state. This occurs between embryonic days 12 and 14 and manifests itself most strongly in the lymphoid-primed multipotent progenitor population, thus pointing to a window of opportunity and a potential cell of origin. However, this state alone is insufficient to generate disease, with the mice succumbing to B cell lymphomas only after a long latency. Future analysis of the molecular details of this pre-leukemic state will shed light on additional events required for progression to acute leukemia.

INTRODUCTION

While many pediatric leukemias have enjoyed significant advances in treatment in recent years that dramatically increase long-term survival rates, infant leukemia associated with the MLL-AF4 fusion continues to have a dismal prognosis. Among infant leukemias, MLL-AF4 is the most frequent translocation and results in an aggressive disease with a very early onset (<1 year of age), characterized by a pro-B acute lymphoblastic leukemia (ALL) phenotype or, in some cases, biphenotypic leukemia (Sanjuan-Pla et al., 2015). Studies on monozygotic twins and the retrospective analysis of blood taken at birth have established that MLL-AF4-associated leukemia has a prenatal origin (Greaves, 2005). Furthermore, the observation that leukemic cells carry no or infrequent additional mutations, together with the early onset, rapid progression, and the fact that it can present itself with ALL or a biphenotypic disease, has led to the suggestion that the cell of origin is a developmentally restricted embryonic/fetal progenitor that does not exist in the adult hematopoietic system (Andersson et al., 2015; Daser and Rabbitts, 2005). It is proposed that this cell has unique properties that might include a more permissive chromatin state and a less restricted differentiation potential, facilitating its transformation.

The in utero origin of MLL-AF4-associated infant leukemia poses a major challenge to the study of this malignancy. For this reason a faithful in vitro or animal model is required to allow analysis of the early changes in the blood system that lead to leukemia development. Such models are also a prerequisite for elucidating the pathogenesis of the disease, as well as testing treatments. A number of different models have been established, which range from transduction of human embryonic stem cells (ESCs) and cord blood cells to the generation of genetic mouse lines, none of which was able to faithfully recapitulate the disease in infant patients (Bueno et al., 2012; Bursen et al., 2010; Chen et al., 2006; Krivtsov et al., 2008; Metzler et al., 2006; Montes et al., 2011). The transduction of human ESCs and cord blood cells with MLL-AF4 did not result in transformation; however, it altered the differentiation path of ESCs, enhancing hemogenic precursors, which were then skewed toward the endothelial lineage (Bueno et al., 2012). By contrast, in cord blood cells, MLL-AF4 caused a slight increase in engraftment potential, myeloid CFU-C output, proliferation, and survival (Montes et al., 2011). Interestingly, while transduction of mouse Lin-Sca1+ cells with MLL-AF4 (albeit at very low transduction efficiencies) had no effect, transduction with the reciprocal fusion AF4-MLL produced pro-B ALL with a long latency (Bursen et al., 2010).

To study disease development in vivo, a number of genetic mouse models have been generated. A straight Mll-AF4 knockin (Chen et al., 2006) and a conditional invertor line (Metzler et al.,

This is an open access article under the CC BY license (http://creativecommons.org/licenses/by/4.0/).

OPEN ACCESS
CellPress

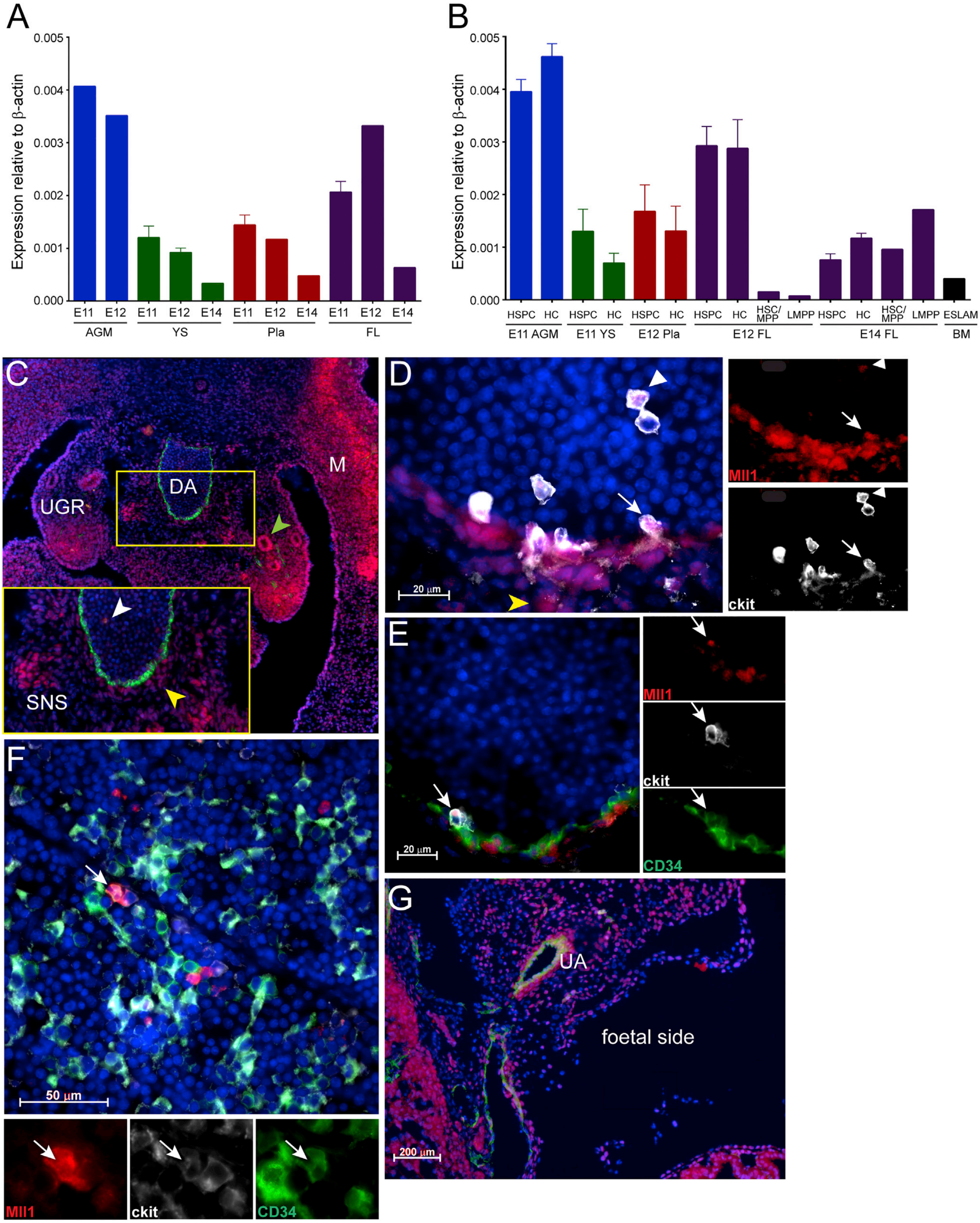

(legend on next page)

2006), in which expression of Mll-AF4 was induced with lymphoid-specific Cre recombinases, both produced more mature B lymphomas with a very long latency. A conditional knockin line, in which Mll-AF4 was induced by Mx1-Cre in adult animals, developed both pre-B ALL and acute myeloid leukemia (AML) with a shorter latency that was still around 150 days (Krivtsov et al., 2008). The reasons for the failure to recapitulate the phenotype of the human disease are unknown; however, they may include the following: (1) additional mutations and/or the presence of both fusion products are required, or (2) the models failed to target the right cell in the right cellular context.

As recent sequencing studies have revealed that MLL-AF4+ infant leukemias do not seem to require any additional mutations apart from the initial translocation (Andersson et al., 2015; Bardini et al., 2011), we decided to concentrate on the second possibility. We used the conditional invertor line (Metzler et al., 2006) and targeted oncogene expression to the first definitive blood cells. We then analyzed how this affected blood development in the embryo and fetus, and we demonstrated that Mll-AF4 enhances lymphoid output and self-renewal in hematopoietic stem cells (HSCs) and immature progenitors during a restricted developmental window. The identification of this window of opportunity and a potential cell of origin now allows us to dissect the molecular details of the initial changes produced by MLL-AF4-induced transformation, which may highlight points of therapeutic intervention.

RESULTS

Mll1 Is Widely Expressed in the Developing Hematopoietic System

Since the expression of the *Mll-AF4* fusion gene is controlled by the regulatory elements of the endogenous murine *Mll1* gene, we analyzed *Mll1* expression in mouse embryonic and fetal hematopoietic tissues, as well as sorted cell populations, by qPCR to narrow down the potential target cells for transformation. There are four major hematopoietic sites during mammalian development (reviewed in Medvinsky et al., 2011 and Mirshekar-Syahkal et al., 2014). The aorta-gonads-mesonephros (AGM) region is the first location in which adult-repopulating HSCs are detected, starting from embryonic day (E)10.5 (Medvinsky and Dzierzak, 1996; Müller et al., 1994). Prior to HSC emergence, the yolk sac provides a variety of primitive and definitive blood cells and progenitors, which are essential for embryonic survival (reviewed in Frame et al., 2013). More recently, the placenta also was described as being a rich source of hematopoietic stem and progenitor cells (Alvarez-Silva et al., 2003; Gekas et al., 2005; Ottersbach and Dzierzak, 2005), with a peak at E12. Cells from all of those sites contribute to the colonization of the fetal liver (FL), which, starting from E12, takes over as the main hematopoietic organ of the fetus.

Analysis of RNA extracted from whole tissues at stages that cover their peaks of hematopoietic activity showed that *Mll1* is expressed in all of the tissues at all time points analyzed, with the highest levels in the AGM and FL (Figure 1A). To look more specifically at the expression of *Mll1* in hematopoietic cells (HCs), we sorted HSC-enriched populations (hematopoietic stem and progenitor cell [HSPC], HSC/multipotent progenitor [MPP], and ESLAM) and all the remaining HCs from these tissues. In addition, we isolated a recently described progenitor that has myeloid and lymphoid potential but lacks erythroid and megakaryocyte capacity (Böiers et al., 2013). This immune-restricted progenitor appears to be the embryonic counterpart of the lymphoid-primed MPP (LMPP) identified by the same group previously (Adolfsson et al., 2005), and it is a prime candidate for the cell of origin for MLL-AF4+ infant leukemia due to its dual lymphoid-myeloid potential (Malouf and Ottersbach, 2013). We found *Mll1* expression in all of these cell types, suggesting a widespread expression of *Mll1* in the hematopoietic system (Figure 1B).

To obtain a better picture of the spatial pattern of *Mll1* expression, we also performed immunohistochemistry with an antibody to Mll1 on embryo sections. Within the AGM, Mll1 was found in circulating HCs in the aortic lumen (white arrowhead in Figures 1C, inset, and 1D), in sub-aortic mesenchymal cells (yellow arrowhead in Figures 1C, inset, and 1D), in the mesonephric duct (green arrowhead in Figure 1C), in cells of the sympathetic nervous system (Figure 1C, inset), and in the tips of the urogenital ridges (Figure 1C). Co-staining with antibodies to ckit and CD34 revealed that Mll1 also is found in cells of intra-aortic clusters, which are believed to be sites of hematopoietic stem and progenitor emergence (white arrows in Figures 1D and 1E). Cells that were positive for all three markers also were found in the E14 fetal liver (Figure 1F). There was widespread expression of Mll1 in the placenta, with cells in and around the major vessel, including the umbilical artery, also being stained (Figure 1G).

Mll-AF4 Expression Does Not Significantly Alter Embryonic/Fetal Blood Composition

To study the effect of MLL-AF4 expression on embryonic and fetal blood cells, we obtained the conditional Mll-AF4 invertor mouse line (Metzler et al., 2006) and crossed it with the Vav-Cre (Stadtfeld and Graf, 2005) or VE-Cadherin (VEC)-Cre line (Chen et al., 2009) (Figure 2A). Vav-Cre targets all of the definitive

Figure 1. Mll1 Displays a Widespread Expression Pattern during Development

(A and B) The qPCR for Mll1 expression was performed on (A) whole tissues (n = 2–6) or (B) sorted HC populations (n = 1–5). HSPC, CD34+ ckit+; HC, CD45+ CD34− CD19−; HSC/MPP, Lin− B220− CD19− ckit+ CD45+ Sca1+ Flt3− IL7R−; LMPP, Lin− B220− CD19− ckit+ CD45+ Sca1+ Flt3+; ESLAM, EPCR+ CD45+ CD150+ CD48−. Error bars indicate SEM.

(C–G) Immunohistochemistry was performed showing Mll1 protein presence in the (C) E11 AGM (Mll1, red; CD34, green; DAPI, blue; inset, enlargement of boxed-in area; green arrowhead, Mll1 expression in mesonephric ducts; white arrowhead, expression in circulating cells; yellow arrowhead, expression in sub-aortic mesenchymal cells; DA, dorsal aorta; M, myotome; SNS, sympathetic nervous system; UGR, urogenital ridges; ×20 objective); (D) E11 dorsal aorta (Mll1, red; ckit, white; DAPI, blue; yellow arrowhead, Mll1 expression in the sub-aortic mesenchyme; white arrowhead, expression in circulating ckit+ cells; white arrow, expression in ckit+ intra-aortic cluster cells; ×100 objective); (E) E11 dorsal aorta; Mll1, red; ckit, white; CD34, green; DAPI, blue; white arrow, expression in ckit+CD34+ intra-aortic cluster cells; ×100 objective); (F) E14 FL (Mll1, red; ckit, white; CD34, green; DAPI, blue; white arrow, cell showing co-expression of all three markers; ×63 objective); and (G) E12 placenta (Mll1, red; CD34, green; DAPI, blue; UA, umbilical artery; ×5 objective).

OPEN
ACCESS
CellPress

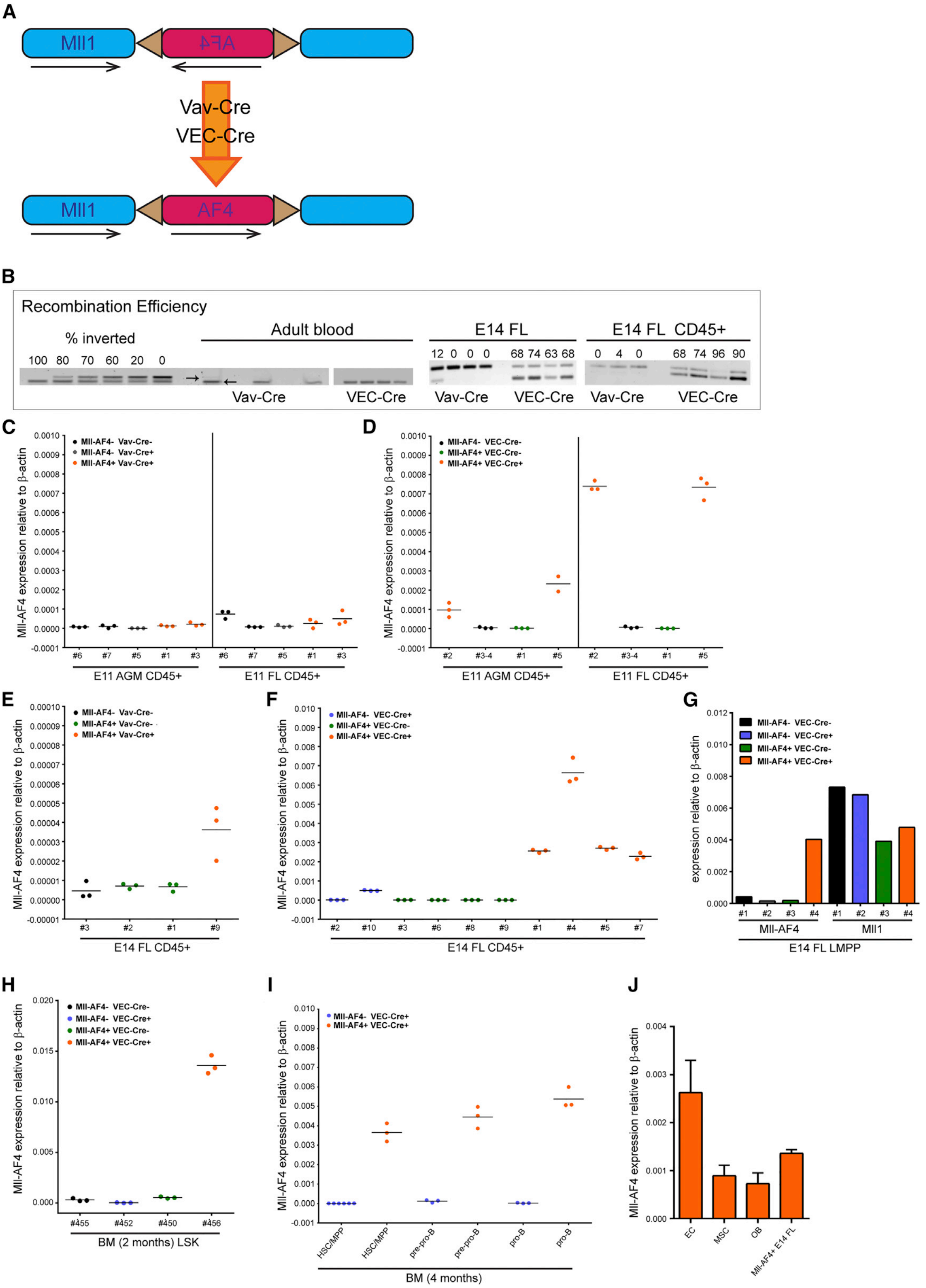

Figure 2. Mll-AF4 Is Expressed following Recombination Mediated by Vav-Cre or VEC-Cre

(A) Schematic diagram shows fusion gene generation.

(B) Recombination efficiencies in E14 FL and adult blood. Standards and adult blood samples have the non-inverted band at the top and a *Myo* band for DNA quantification at the bottom; E14 FL samples have the non-inverted band at the top and the inverted band at the bottom, which allows for direct quantification.

(legend continued on next page)

HCs as early as E11, while VEC-Cre targets the precursors of definitive HCs, the hemogenic endothelium (Chen et al., 2009). While recombination efficiency in adult blood was equally high with Vav-Cre and VEC-Cre (both reaching 100%), it was much higher in total and CD45+ E14 FL cells following VEC-Cre-mediated recombination compared with Vav-Cre-mediated recombination (Figure 2B). For this reason, we concentrated our analysis predominantly on tissues derived from Mll-AF4 × VEC-Cre crosses.

We also checked for the expression of the fusion transcript in CD45+ cells and were able to detect expression following VEC-Cre induction as early as E11 in the AGM and at even higher levels in the FL, while it was virtually non-detectable in those tissues following Vav-Cre induction (Figures 2C and 2D). Transcript levels increased in the E14 FL and remained markedly higher with VEC-Cre as compared with Vav-Cre (Figures 2E and 2F). In fact, *Mll-AF4* expression in sorted cells from the E14 FL following VEC-Cre recombination reached levels comparable to the endogenous *Mll1* gene from the remaining allele (Figure 2G). Specific progenitor populations in the bone marrow (BM) of young mice also were analyzed, and they revealed Mll-AF4 expression in HSC-enriched populations (HSC/MPP), pre-pro-B and pro-B cells, the exact cell types implicated in Mll-AF4+ leukemia (Figures 2H and 2I). The stromal microenvironment is increasingly recognized as a contributing factor to the development of hematological malignancies (Krause and Scadden, 2015). To determine whether VEC-Cre-induced recombination leads to fusion gene expression in the microenvironment, defined BM stromal cell populations (Schepers et al., 2013) were sorted and analyzed for *Mll-AF4* transcript expression by qPCR. The highest expression was detected in endothelial cells, with only low signals in mesenchymal stromal cells and osteoblasts (Figure 2J).

To determine if Mll-AF4 expression altered blood production in the embryo, specific hematopoietic populations were analyzed by flow cytometry at different developmental time points. E11 AGM HSCs are enriched within the CD41intCD34+ckit+, while E11 yolk sac (YS) HSCs are within the CD34+ckit+ population; however, their percentages did not significantly differ between the different genotypes (Figures S1A and S1B). As CD19 is virtually undetectable on the surface of E11 and E12 cells, we restricted ourselves to B220 expression for the detection of B cells, demonstrating that the percentage of B220+ cells was unaltered in the AGM (Figure S1C). At E12, the FL begins to take over as the most important hematopoietic organ in the fetus. We therefore analyzed CD34+ckit+, B220+, and LMPP numbers in E12 FL samples, and we also found these to be unchanged (Figures S1D–S1F). Further enrichment for HSCs can be achieved in the E14 FL with the EPCR+CD45+ CD150+CD48− (ESLAM) (Kent et al., 2009) phenotype. The percentages of these populations were again unaffected by the presence of Mll-AF4 (Figure S1G), as were those of B cells and LMPPs (Figures S1H and S1I).

Mll-AF4 Expression Is Associated with Modest Myeloid Expansion

To assess the effect of Mll-AF4 expression on hematopoietic progenitor function, dissociated FL cells from Mll-AF4 × Vav-Cre and Mll-AF4 × VEC-Cre crosses were plated in methylcellulose assays that promoted the growth of myeloid colonies. Using E12 FL cells, a significant increase in total colony numbers was noted upon Mll-AF4 expression (Figure 3A). Furthermore, when the cells were collected from the plates and analyzed for CD11b and Gr1 expression, an expansion of CD11b+Gr1+ cells was demonstrated (Figure 3B). A similar effect was observed with E14 FL cells, albeit slightly less prominent (Figures 3C and 3D). To test the myeloid potential of specific cell populations, HSCs/MPPs and LMPPs were sorted from FL cells resulting from Mll-AF4 × VEC-Cre crosses and plated in methylcellulose. At both E12 and E14, a 2-fold increase in total colonies was noted from HSCs/MPPs expressing Mll-AF4, while only colony-forming unit (CFU)-granulocyte, monocyte (GM) output from LMPPs was significantly increased (Figures 3E and 3F).

MLL fusions may confer properties of self-renewal on progenitor populations in vitro, which was tested through serial replating in methylcellulose assays under myeloid conditions. E14 FL HSCs/MPPs from the three controls failed to replate after the third round, while Mll-AF4-expressing HSCs/MPPs were able to give rise to colonies for another two rounds, at which point the cells in the colonies had a CD45+ ckit+ CD11b− Gr1− phenotype. After the fifth round they also exhausted, indicating that full transformation had not been achieved (Figure 3G). We also plated adult BM cells in myeloid colony-forming assays, but we saw no difference in colony numbers between Mll-AF4-expressing cells and Cre controls (Figure 3H); and, unlike FL cells, Mll-AF4-expressing BM HSCs/MPPs did not replate after the second round (Figure 3I).

(C and D) *Mll-AF4* expression by qPCR in sorted CD45+ cells from E11 AGM and FL of indicated genotypes following (C) Vav-Cre recombination or (D) VEC-Cre recombination. Numbers on x axis stand for individual embryos.

(E and F) *Mll-AF4* expression by qPCR in CD45+ cells from E14 FL of indicated genotypes following (E) Vav-Cre recombination or (F) VEC-Cre recombination. Numbers on x axis stand for individual embryos.

(G) *Mll-AF4* and *Mll1* expression by qPCR in sorted E14 FL LMPP (Lin− B220− CD19− ckit+ CD45+ Sca1+ Flt3+) cells of indicated genotypes following VEC-Cre recombination is shown.

(H) *Mll-AF4* expression by qPCR in Lin−Sca1+ckit+ (LSK) cells sorted from adult BM of indicated genotypes following VEC-Cre recombination. Numbers on x axis stand for individual mice.

(I) *Mll-AF4* expression by qPCR in sorted BM populations of indicated genotypes following VEC-Cre recombination is shown (HSC/MPP, Lin− CD93+ CD24int CD43+ B220− CD19− ckit+ IL7R−; pre-pro-B, Lin− CD93+ CD24+ CD43+ B220+ CD19− ckitint IL7Rint; and pro-B, Lin− CD93+ CD24+ CD43+ B220+ CD19+ ckit+ IL7R+).

(J) Endothelial cells (ECs, Lin− CD45− CD31+ Sca1+), mesenchymal stromal cells (MSCs; Lin− CD45− CD31− Sca1+ CD51+), and osteoblasts (OBs; Lin− CD45− CD31− Sca1− CD51+) were sorted from Mll−AF4+ VEC−Cre+ adult BM and analyzed for *Mll-AF4* transcript expression by qPCR. Mll-AF4+ VEC-Cre+ E14 total FL cell cDNA was included as a positive control (n = 3). Error bars indicate SEM. See also Figure S1.

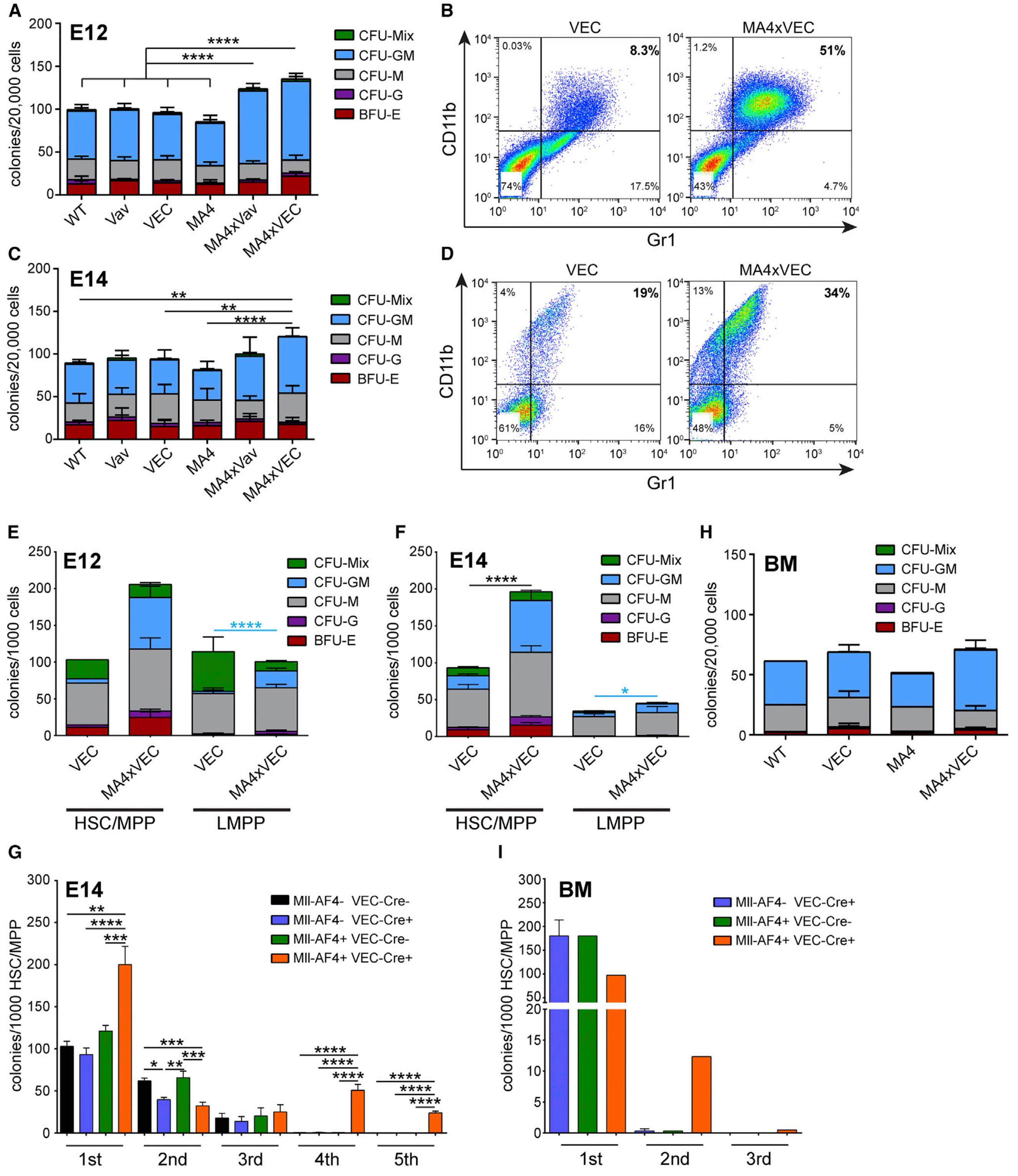

Figure 3. Mll-AF4 Expression Leads to a Mild Myeloid Expansion

(A) Myeloid colony formation from E12 FL cells of the indicated genotypes is shown (n = 4–5).

(B) CD11b and Gr1 expression on cells isolated from E12 FL myeloid colony plates of the indicated genotypes is shown.

(C) Myeloid colony formation from E14 FL cells of the indicated genotypes is shown (n = 3–7).

(D) CD11b and Gr1 expression on cells isolated from E14 FL myeloid colony plates of the indicated genotypes is shown.

(legend continued on next page)

Mll-AF4 Confers Enhanced Lymphoid Potential

MLL-AF4+ infant leukemia is predominantly associated with a pro-B ALL phenotype. We therefore assessed the influence of Mll-AF4 expression on B lymphoid potential in methylcellulose assays. B lymphoid potential can be detected in the AGM as early as E9.5 with a peak at E11 (Godin et al., 1999). When plated directly in methylcellulose, AGM cells produced very few B cell colonies; however, they were significantly enhanced when Mll-AF4 was expressed (Figure 4A). Control E11 FL cells produced equally low numbers of lymphoid colonies, but this was enhanced by >7-fold in the presence of Mll-AF4 (Figure 4B). This obvious difference between control plates and Mll-AF4+ plates was very easily observable by eye (Figure 4C). In addition, the colonies produced in the presence of Mll-AF4 tended to be much larger (Figure 4D, bottom) than control colonies (Figure 4D, top), indicating that Mll-AF4 not only increases the number of progenitors but also enhances their proliferative capacity. The cells collected from the Mll-AF4+ plates had a B220+CD19+ CD11b−Ter119− cell surface phenotype (Figure 4G).

The amplification of B lymphoid colonies was even more significant at E12, reaching 7-fold and 8-fold following Vav-Cre and VEC-Cre induction, respectively, with a significant difference between Vav-Cre and VEC-Cre (Figure 4E). To be able to link this to a specific cell of origin, HSC/MPP and LMPP populations were isolated from E12 FL cells and plated in lymphoid assays. The results suggest that LMPPs are the major contributors to the enhanced B lymphoid output, with a 25-fold increase upon Mll-AF4 expression (Figure 4F). Similar effects were observed with E14 total FL and sorted cell populations (Figures 4H and 4I). The fold differences between Cre controls and Mll-AF4-expressing cells were 4.5- and 7-fold for Vav-Cre and VEC-Cre, respectively, with total E14 FL cells. We also collected the cells from the colony plates and analyzed their surface phenotype. This revealed that Mll-AF4 expression causes an accumulation of cells with a pro-B cell phenotype (Figure 4J), representing the stage at which differentiation is blocked in patients.

A significantly increased B lymphoid output also was observed from Mll-AF4-expressing BM cells, albeit on a smaller scale (3-fold; Figure 4K), supporting the existence of a window of opportunity at E12–14. This difference in enhanced B lymphoid output between FL and BM cells is particularly striking when sorted HSC/MPP and LMPP populations are compared (compare Figures 4I and 4L). The main contributor to increased B lymphoid colonies from BM cells is likely the common lymphoid progenitor (CLP) population, but not more differentiated cells (Figure 4M). FL cells showed limited replating potential in lymphoid conditions (Figure 4N). Only Mll-AF4-expressing cells were able to give rise to secondary colonies upon replating, and these failed to produce colonies in a further round.

MLL is an H3K4 methyltransferase that interacts with its target genes via its N terminus and carries the methyltransferase activity in its C terminus. As a result of leukemogenesis-associated translocations, the C terminus of MLL is replaced with the C terminus of a number of fusion partners that share the ability to interact with the super-elongation complex (SEC) and lead to aberrant expression of MLL target genes (reviewed in Krivtsov and Armstrong, 2007 and Slany, 2009). Another hallmark of MLL fusion oncogenes is their ability to recruit the H3K79 methyltransferase DOT1L to MLL target genes, which results in characteristically high levels of H3K79 methylation in MLL-rearranged leukemias and makes DOT1L an attractive therapeutic target (Daigle et al., 2011; Krivtsov et al., 2008).

To test whether these mechanisms underlie the hematopoietic changes we observed in the fetus, a number of small molecule inhibitors were employed. I-BET151 is a recently described inhibitor of the BET family of proteins that mediates the interaction of SEC with chromatin (Dawson et al., 2011). The disruption of this interaction with I-BET151 was shown to interfere with leukemogenesis induced by MLL fusions. We therefore added this inhibitor to B lymphoid assays, which significantly reduced the number of colonies produced by Mll-AF4-expressing FL cells (Figure S2A). We next tested two different inhibitors of DOT1L, SGC0946 and EPZ-5676 (Daigle et al., 2013; Yu et al., 2012), and we observed that these also significantly inhibited the growth of B lymphoid colonies from Mll-AF4+ FL cells (Figure S2A). None of the inhibitors displayed any effect on control colony formation (Figure S2B), although B lymphoid colony formation from control cells was low. We therefore repeated the inhibitor experiments with adult BM cells from mice that do not yet show any obvious signs of disease (Figures S2C and S2D). The effects of all three inhibitors were still stronger on Mll-AF4-expressing cells, although I-BET151 displayed some degree of toxicity on normal cells. These results indicate that the activity of the Mll-AF4 fusion, via aberrant SEC and DOT1L recruitment, is responsible for the expansion of lymphoid progenitors in our model.

Mll-AF4 Endows HSCs with Enhanced Repopulation and Self-Renewal Potential

Many leukemia oncogenes can confer self-renewal ability. To assess the effect of Mll-AF4 on embryonic HSCs, hematopoietic tissues from different developmental time points were transplanted in a non-competitive setting. The first tissue in which adult-repopulating HSCs are detectable is the AGM region, with HSC numbers peaking at E11 (Kumaravelu et al., 2002; Medvinsky and Dzierzak, 1996). Interestingly, Mll-AF4 appears to have a negative impact on emerging HSCs, as the fewest number of mice were repopulated with Mll-AF4+ AGM cells

(E) Myeloid colony formation from sorted E12 FL HSC/MPP (Lin− B220− CD19− ckit+ CD45+ Sca1+ Flt3− IL7R−; n = 2, VEC; n = 4, MA4xVEC) and LMPP (Lin− B220− CD19− ckit+ CD45+ Sca1+ Flt3+) cells is shown (n = 3 for both genotypes).

(F) Myeloid colony formation from sorted E14 FL HSC/MPP (n = 5, VEC; n = 7, MA4xVEC) and LMPP cells is shown (n = 5, VEC; and n = 4, MA4xVEC).

(G) Replating potential of E14 FL HSC/MPP of indicated genotypes in myeloid conditions is shown (n = 3–5).

(H) Myeloid colony formation from total adult BM cells of indicated genotypes is shown (n = 1, WT; n = 3, VEC; n = 2, MA4; and n = 4, MA4xVEC).

(I) Replating potential of adult BM HSC/MPP of indicated genotypes in myeloid conditions is shown (n = 3, VEC; n = 1, MA4; and n = 2, MA4xVEC).

Statistical analysis was performed using a one-way ANOVA with Tukey's multiple comparisons test (****$p < 0.0001$, ***$p < 0.001$, **$p < 0.01$, and *$p < 0.05$). Error bars indicate SEM.

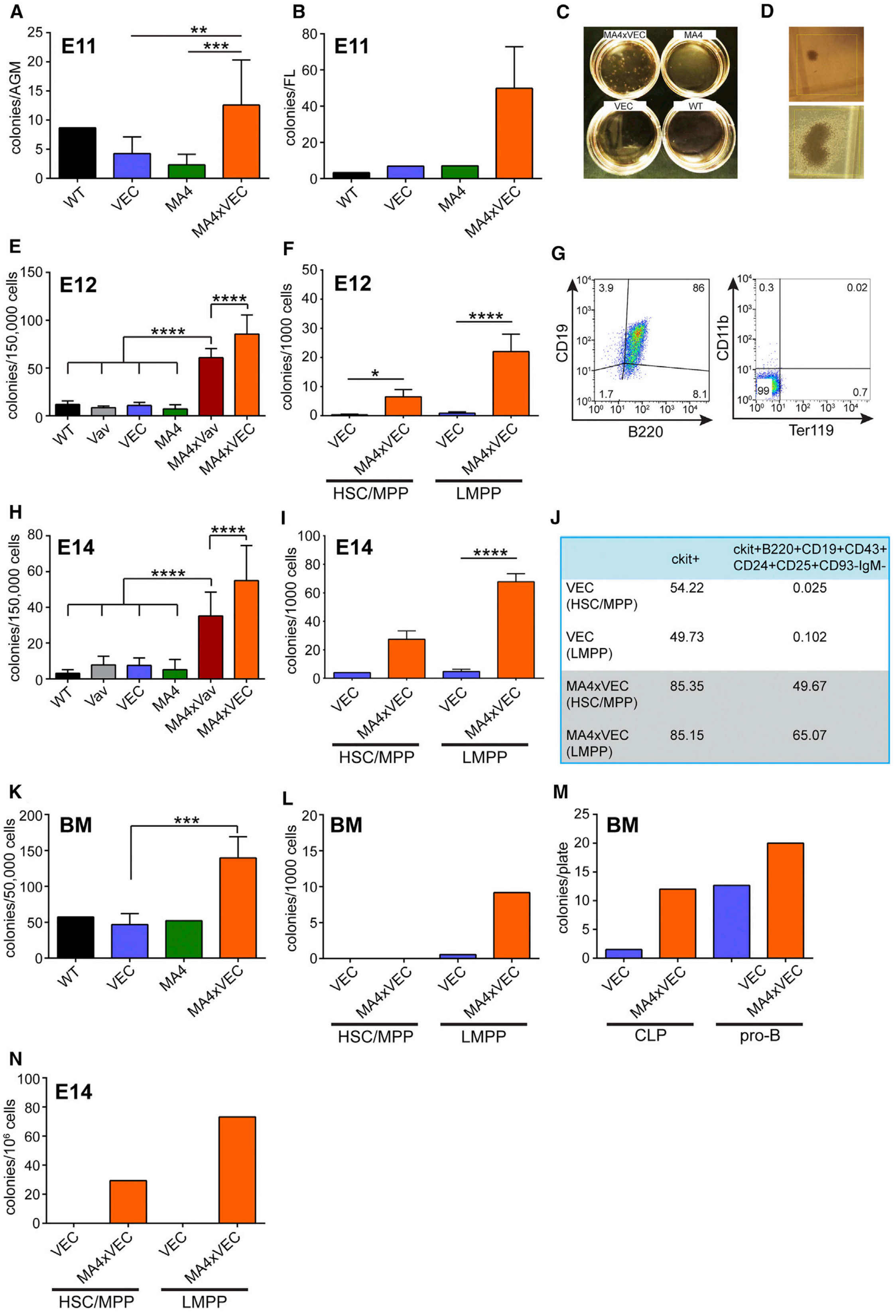

(legend on next page)

with the lowest level of chimerism (Figure 5A). Before the establishment of the FL as the main hematopoietic site, the HSC pool is expanded in the placenta (Gekas et al., 2005; Ottersbach and Dzierzak, 2005). At E12, the peak of expansion, Mll-AF4 expression had a positive effect on placental HSCs, resulting in all mice being repopulated and overall reconstitution levels being higher (Figure 5B). The recipient mice also presented with a higher white blood cell (WBC) count (Figure 5C). After E12, the FL becomes the main hematopoietic organ (Gekas et al., 2005; Kumaravelu et al., 2002). Mice transplanted with E12 FL cells expressing Mll-AF4 showed the highest level of donor chimerism in the peripheral blood (>90%), while mice injected with cells carrying the non-inverted allele had reduced donor contribution (Figure 5D). The latter is most likely due to the haploinsufficiency of wild-type (WT) Mll1 (Ernst et al., 2004), a trend that was also observed with the E12 placenta. Interestingly, within the donor population, myeloid contribution was increased while T cells were reduced (Figure 5E).

To assess the impact on self-renewal, secondary transplantations were performed. When BM cells of primary recipients of E12 FL cells were injected into secondary recipients, all mice receiving Mll-AF4-expressing cells were repopulated as compared with 50% of recipients of Cre-only cells (Figure 5F). There was also a dramatic difference in donor chimerism, overall suggesting that Mll-AF4 imparts enhanced self-renewal capacity. Secondary recipients of Mll-AF4+ cells also had higher WBC counts (Figure 5G).

Mice receiving E14 FL Mll-AF4+ cells again showed the highest level of reconstitution (around 70%) compared with Cre-only and Mll-AF4 non-inverted cells, the latter of which were again impaired in their repopulation ability (Figure 5H). Donor contribution to the T cell lineage was again significantly reduced, and there was also a trend toward higher B and myeloid cell contribution (Figure 5I). In secondary transplantations, all recipients of E14 FL Mll-AF4-expressing cells were repopulated as compared with 36% of recipients of Cre-only cells, and there was again a significant difference in donor chimerism (Figure 5J) and a trend toward higher WBC counts (Figure 5K). Donor cells also produced more B220+CD19+ compared to Cre-only cells (data not shown). Mll-AF4-expressing E12 placenta cells also performed better in secondary transplants than control cells (Figure 5L), with a slight increase in WBCs (Figure 5M). Furthermore, unlike Cre-only cells, which did not reconstitute at all, Mll-AF4-expressing cells were able to fully reconstitute tertiary recipients, producing consistently higher levels of chimerism (Figure 5N) and higher WBC counts (Figure 5O).

Mll-AF4 Expression in Embryonic Cells Causes B Cell Lymphoma with a Long Latency

To determine whether targeting of Mll-AF4 expression to early hematopoietic progenitors results in the development of leukemia comparable to the disease found in infant patients, offspring from Mll-AF4 × Vav-Cre and Mll-AF4 × VEC-Cre crosses were monitored for disease development. Mice from these crosses were born at normal Mendelian ratios and had no obvious phenotype apart from a trend toward a lower body weight (data not shown). All of the mice that expressed the fusion gene died significantly earlier than their control littermates, however, only after a long latency (Figure 6A). The median survival was 437 days for Mll-AF4+ VEC-Cre+ mice and 556 days for Mll-AF4+ Vav-Cre+ mice, which is similar to what had been observed previously when Mll-AF4 expression was targeted to the lymphoid lineage (Metzler et al., 2006). Mll-AF4+ VEC-Cre+ mice seem to die slightly earlier than Mll-AF4+ Vav-Cre+ mice; however, the difference was not significant.

The majority of the mice (74%) presented with B cell lymphomas, with three mice showing a T cell disease and three a lymphoproliferative disease (Table 1). Almost all of the mice displayed pronounced splenomegaly and liver infiltration, and the majority also developed mesenteric tumors (Table 1; Figures 6B–6D). A number of the mice also presented with mediastinal tumors and kidney infiltration, but the BM and peripheral blood were only rarely affected (Table 1). This disease phenotype was similar to that observed in other MLL-AF4 mouse models

Figure 4. Mll-AF4 Expression Leads to Increased B Lymphoid Output

(A) B lymphoid colony formation from E11 AGM cells of the indicated genotypes is shown (n = 1, WT; n = 4, VEC; n = 3, MA4; and n = 4, MA4xVEC).

(B) B lymphoid colony formation from E11 FL cells of the indicated genotypes is shown (n = 1, WT; n = 2, VEC; n = 1, MA4; and n = 4, MA4xVEC).

(C) B lymphoid colony assay plates are shown.

(D) Representative examples show the type of B lymphoid colonies found on control plates (top) and plates that contain Mll-AF4-expressing cells (bottom), at the same magnification (×4).

(E) B lymphoid colony formation from E12 FL cells of the indicated genotypes is shown (n = 5).

(F) B lymphoid colony formation from sorted E12 FL HSC/MPP (Lin− B220− CD19− ckit+ CD45+ Sca1+ Flt3− IL7R−) and LMPP (Lin− B220− CD19− ckit+ CD45+ Sca1+ Flt3+) cells is shown (n = 3–4).

(G) Cell surface phenotype of cells collected from colonies is shown.

(H) B lymphoid colony formation from E14 FL cells of the indicated genotypes is shown (n = 3–8).

(I) B lymphoid colony formation from sorted E14 FL HSC/MPP (n = 2, VEC; and n = 4, MA4xVEC) and LMPP cells (n = 5, VEC; and n = 5, MA4xVEC) is shown.

(J) Percentage of cells with a ckit+ or pro-B phenotype collected from B lymphoid plates that had been seeded with sorted (HSC/MPP or LMPP) E14 FL cells of indicated phenotypes is shown.

(K) B lymphoid colony formation from BM cells of the indicated genotypes (n = 1, WT; n = 3, VEC; n = 2, MA4; and n = 4, MA4xVEC) is shown.

(L) B lymphoid colony formation from sorted adult BM HSC/MPP (Lin− B220− CD19− ckit+ CD45+ Sca1+ Flt3− IL7R−) and LMPP (Lin− B220− CD19− ckit+ CD45+ Sca1+ Flt3+) cells (n = 3, VEC; and n = 2, MA4xVEC) is shown.

(M) B lymphoid colony formation from sorted adult BM CLP (Lin− CD93+ CD24int CD43+ B220− CD19− ckit+ IL7R+) and pro-B (Lin− CD93+ CD24+ CD43+ B220+ CD19+ ckit+ IL7R+) cells (n = 2, VEC; and n = 1, MA4xVEC) is shown.

(N) Replating potential of E14 FL HSC/MPP and LMPP of indicated genotypes in lymphoid conditions. Results are shown after one round of replating (n = 2).

Statistical analysis was performed using a one-way ANOVA with Tukey's multiple comparisons test (****p < 0.0001, ***p < 0.001, **p < 0.01, and *p < 0.05). Error bars indicate SEM. See also Figure S2.

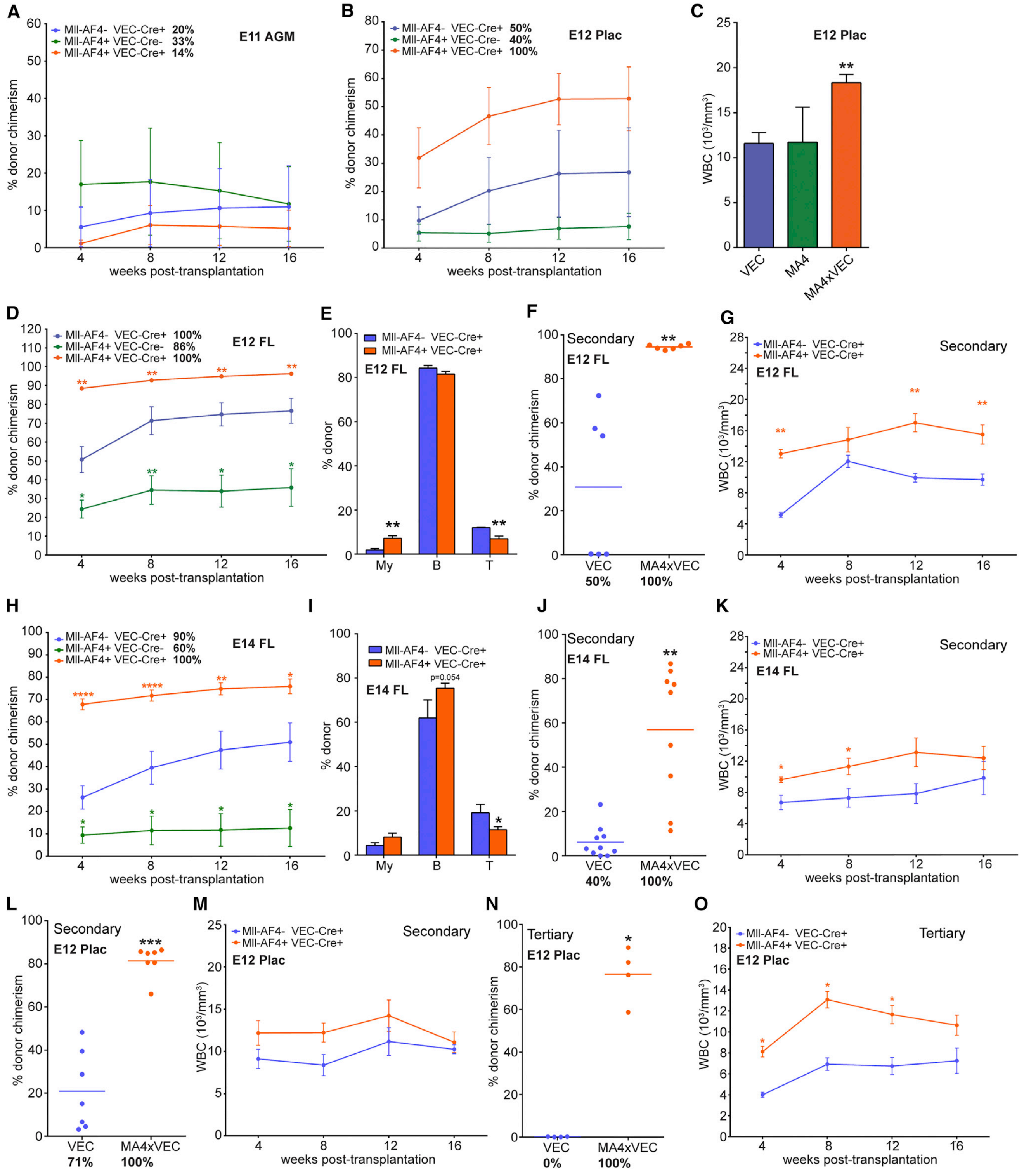

Figure 5. Mll-AF4-Expressing HSCs Have Higher Repopulation and Self-Renewal Activity

(A and B) Average donor contribution to the peripheral blood of all recipients of (A) E11 AGM cells (recipients n = 5, VEC; n = 2, MA4; n = 7, MA4xVEC) or (B) E12 placenta cells (recipients n = 6, VEC; n = 5, MA4; and n = 6, MA4xVEC) with the indicated genotypes. Percentages of mice positive for repopulation are stated next to the genotypes.

(C) White blood cell counts of mice reconstituted with E12 placenta cells at 16 weeks are shown.

(legend continued on next page)

(Chen et al., 2006; Metzler et al., 2006; Tamai et al., 2011). We analyzed some mice at a much earlier time point when they were still asymptomatic, and we observed initial signs of the disease, i.e., splenomegaly and liver infiltration; however, disease progression seems to be slow enough for these mice to survive for a considerable time longer.

To ascertain if the primary disease is transplantable, we dissociated the enlarged spleen of a diseased mouse (Figure 6D), which usually contained a strong B cell and T cell component (Figure 6E), and we injected the cells into secondary recipients. Tumors did indeed develop in secondary recipients (Figure 6F), which again had pronounced B and T cell components (Figure 6G). Interestingly, donor cells contributed both to the B and T cell pools in the tumor (Figure 6H), while the recipient almost exclusively contributed to the T cell component (Figure 6I). These likely represent infiltrating reactive T cells that also have been observed by others (Metzler et al., 2006). Overall, 98% of the B cells were donor derived while 64% of the T cells came from the donor and 36% from the recipient (Figure 6J).

Some of the mice that had been transplanted with Mll-AF4-expressing fetal tissue to assess HSC function (Figure 5) also succumbed to hematological malignancies with a shorter latency than non-transplanted mice (Table S1). They all showed splenomegaly to some degree as well as myeloid expansion. Involvement of the liver and mesenteric and mediastinal tumors also were observed in some cases. The strongest effect was again observed in recipients of E12 FL, with the highest number of sick mice and the strongest disease phenotype. One of the secondary recipients also developed the disease and with a slightly shorter latency than the primary recipients.

DISCUSSION

In this study, we targeted the expression of Mll-AF4 to the first definitive HCs that emerge during embryogenesis as well as their precursors, the hemogenic endothelium, and we studied how this affected hematopoietic development. Phenotypically, blood development seemed largely unaffected with no major changes to the percentages of HSPCs and mature blood cell types. This is in contrast to the hematopoietic impairment that was observed following virally driven expression of MLL-AF4 in hemogenic precursors derived from ESCs (Bueno et al., 2012). However, functional assays revealed that the expression of Mll-AF4 imparted greatly enhanced B lymphoid potential on HSCs/MPPs and LMPPs at a time point during development when there is hardly any B lymphoid output from WT cells in the absence of stromal support (i.e., in methylcellulose assays). Considering that the disease in infants manifests itself as pro B-ALL in the majority of cases, this is highly significant and concurs with our observation of an expansion of cells with a pro-B phenotype. Although myelopoiesis was only mildly affected, the self-renewal of HSCs was greatly increased, again in keeping with the known role of MLL-fusion proteins in malignant self-renewal.

Our results also provide evidence for the existence of the previously suggested developmental window of opportunity for this disease to arise, as the strongest effect was observed with E12–E14 tissues, mainly the FL. Enhancement of myelopoiesis and repopulation activity was strongest in E12 fetal tissues, while the increase in B lymphoid potential was most pronounced at E14. Most importantly, the expansion of the pro-B cell stage was not observed in adult Mll-AF4-expressing BM cells under lymphoid-plating conditions. Furthermore, the majority of transplanted mice that succumbed to hematological disease had been injected with E12 FL cells within this window. This observation was not due to these mice having higher repopulation levels, since an increase in the injected dose of E14 FL cells by more than 100-fold did not result in more mice getting sick and did not accelerate the disease (data not shown). Our data also point to the LMPP as a potential cell of origin, as it consistently gave the highest response to Mll-AF4 expression in lymphoid assays and in the expansion of pro-B cells, although such an effect, albeit on a smaller scale, was already visible in the HSC/MPP population.

The changes we observed—enhanced B lymphoid potential, expansion of pro-B cells, and increased self-renewal—may be reasonably described as the pre-leukemic state for this disease. Having been able to capture this state, which is inaccessible in human patients, now allows us to study in more detail the early molecular changes induced by the expression of the fusion gene. It also might provide some indication as to what is missing

(D) Donor contribution to the peripheral blood of recipients of E12 FL cells with the indicated genotypes (recipients n = 5, VEC; n = 7, MA4; and n = 7, MA4xVEC). Percentages of mice positive for repopulation are stated next to the genotypes.
(E) Contribution to individual lineages at 16 weeks is shown.
(F) Donor contribution to the peripheral blood of secondary recipients of E12 FL cells at 16 weeks (recipients n = 6, VEC; and n = 6, MA4xVEC). Dots represent individual mice and the total percentage of repopulated mice is given underneath.
(G) White blood cell counts of secondary recipients of E12 FL cells are shown.
(H) Donor contribution to the peripheral blood of recipients of E14 FL cells with the indicated genotypes (recipients n = 11, VEC; n = 5, MA4; and n = 14, MA4xVEC). Percentages of mice positive for repopulation are stated next to the genotypes.
(I) Contribution to individual lineages at 16 weeks is shown.
(J) Donor contribution to the peripheral blood of secondary recipients of E14 FL cells at 16 weeks (recipients n = 11, VEC; and n = 10, MA4xVEC). Dots represent individual mice and the total percentage of repopulated mice is given underneath.
(K) White blood cell counts of secondary recipients of E14 FL cells are shown.
(L) Donor contribution to the peripheral blood of secondary recipients of E12 placenta cells at 16 weeks (recipients n = 7, VEC; and n = 7, MA4xVEC). Dots represent individual mice and the total percentage of repopulated mice is given underneath.
(M) White blood cell counts of secondary recipients of E12 placenta cells are shown.
(N) Donor contribution to the peripheral blood of tertiary recipients of E12 placenta cells at 16 weeks (recipients n = 4, VEC; and n = 4, MA4xVEC). Dots represent individual mice and the total percentage of repopulated mice is given underneath.
(O) White blood cell counts of tertiary recipients of E12 placenta cells are shown.
Statistical analysis was performed using a Mann-Whitney test (****p < 0.0001, ***p < 0.001, **p < 0.01, and *p < 0.05). Error bars indicate SEM. See also Table S1.

OPEN
ACCESS
CellPress

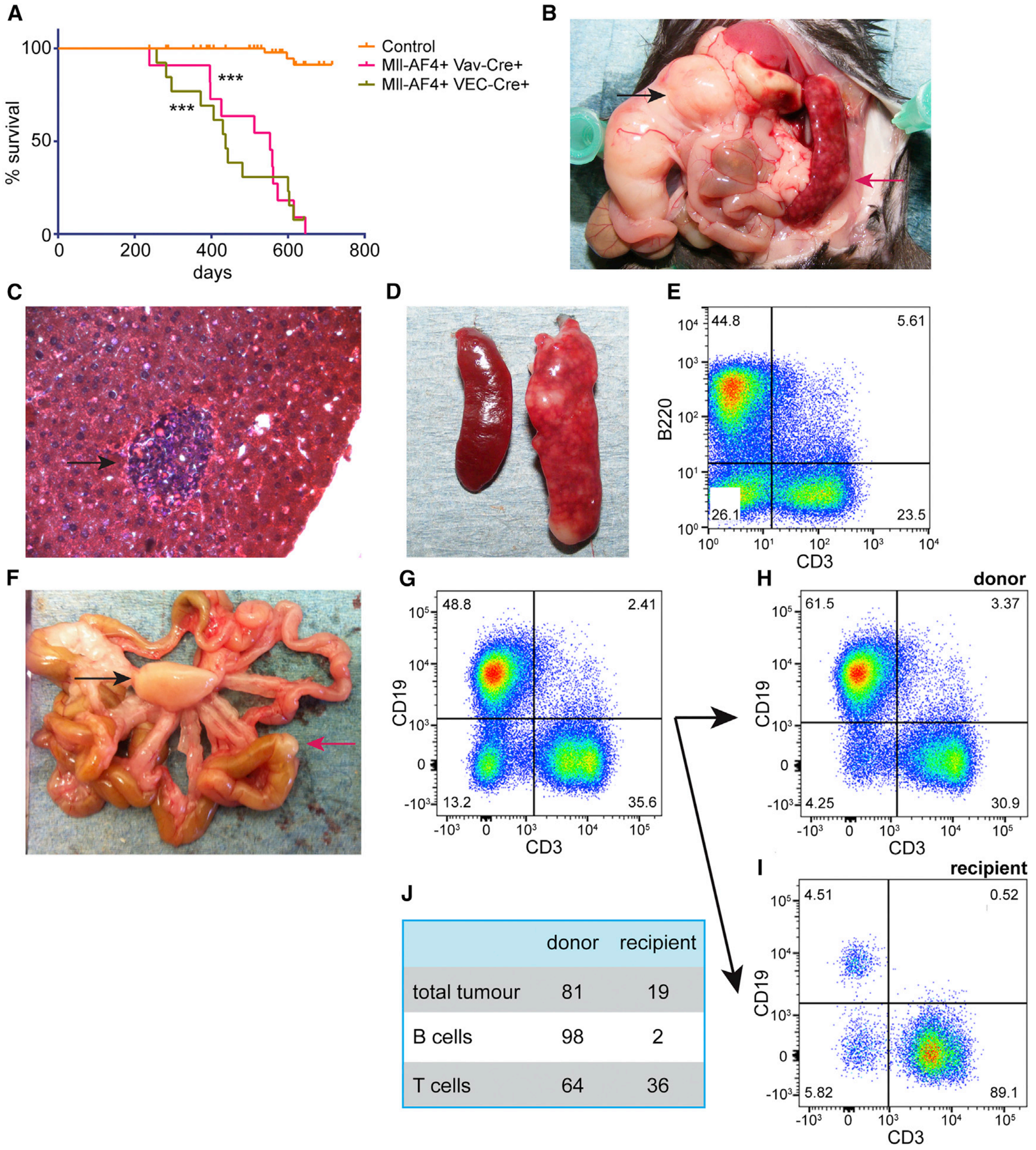

Figure 6. Mll-AF4 Expression Causes B Lymphomas

(A) Kaplan-Meier survival plot. The control curve includes the pooled data from littermates with a WT, VEC-Cre only, Vav-Cre only, and Mll-AF4+Cre-genotypes (***$p < 0.001$).

(B) Diseased mouse with a mesenteric tumor (black arrow) and an enlarged spleen (red arrow) is shown.

(C) H&E-stained section of a diseased liver shows malignant cell infiltration (arrow).

(D) Example shows splenomegaly with the enlarged spleen of an Mll-AF4-expressing mouse on the right and a control spleen on the left.

(E) Diseased spleens contain strong B and T cell components.

(F) Secondary mesenteric (black arrow) and bowl (red arrow) tumors following injection of diseased spleen cells into secondary recipients are shown.

(G–I) B and T cell components in the (G) mesenteric tumor, broken down into (H) donor and (I) recipient, are shown.

(J) Percentages of tumor cell populations that are derived from donor and recipient are shown.

in our model that would convert this pre-leukemic state during that window of opportunity into a short-latency, acute leukemia akin to the disease found in infant patients. The late-onset B lymphomas that result from our experimental approach are similar to the models generated by others (Chen et al., 2006; Metzler et al., 2006; Tamai et al., 2011), and they suggest that we are still missing a key component(s) that drives leukemogenesis.

Table 1. Summary of Disease Phenotypes in Mll-AF4-Expressing Mice

Mouse ID	Splenomegaly	Mesenteric Disease	Mediastinal Disease	BM	Peripheral Blood	Liver	Disease
Mll-AF4 × Vav-Cre (Median Survival 556 Days)							
3809	√	–	√	√	–	√	T cell lymphoma
4468	√	√	–	–	–	√	B cell lymphoma
4475	√	–	–	–	–	√	B cell lymphoma
4649	√	√	–	–	–	√	B cell lymphoma
4650	√	√	–	–	–	√	B cell lymphoma
5244	√	√	–	–	–	–	B cell lymphoma
5250	√	–	–	–	–	√	B cell lymphoma
5909	√	–	–	–	–	√	B cell lymphoma
5911	√	√	–	√	√	√	T cell lymphoma
Mll-AF4 × VEC-Cre (Median Survival 437 Days)							
6357	√	–	–	–	–	√	lymphoproliferative disorder
6703	√	√	–	–	–	√	lymphoproliferative disorder
6705	√	√	–	–	–	√	B cell lymphoma
6707	√	√	–	–	–	√	B cell lymphoma[a]
6709	√	√	–	–	–	√	B cell lymphoma
6713	√	–	–	–	√	√	lymphoproliferative disorder
6809	√	√	–	–	–	√	B cell lymphoma
6810	√	√	–	–	–	√	B cell lymphoma
7060	√	√	–	–	–	√	B cell lymphoma
7063	√	√	√	–	–	√	B cell lymphoma
7737	√	√	√	–	–	√	T cell lymphoma
7739	√	√	√	–	–	√	B cell lymphoma
8945	√	√	–	–	–	–	B cell lymphoma
0062	√	–	–	–	–	√	B cell lymphoma

[a]Transplanted.

Several suggestions as to the identity of this factor have been made and include failure to target the correct cell of origin. By using VEC-Cre to drive Mll-AF4 expression, all of the probable target cells for the initial transformation should have been covered. It has been shown that VEC-Cre labels endothelial cells and circulating blood cells within the yolk sac as early as E8.5 and in the dorsal aorta by E9.5 (Alva et al., 2006; Chen et al., 2009). The two major definitive progenitors that are present after E8.5, the LMPP and the erythroid-myeloid progenitor (EMP), also have been suggested to have a VE-Cadherin+ hemogenic endothelial origin (Böiers et al., 2013; Frame et al., 2013; McGrath et al., 2015). B-1 B cells predominate during fetal development, and there is evidence that they can initiate B-ALL (Montecino-Rodriguez et al., 2014). By crossing VEC-Cre with a ROSA26-red fluorescent protein (RFP) line and analyzing RFP expression in E14 FL B-1 B cells, we found more than 50% of B-1 B cells to be labeled by VEC-Cre (data not shown). Taken together, these data suggest that our approach targeted all the relevant definitive hematopoietic populations. We also initiated Mll-AF4 expression in the germline by crossing the Mll-AF4 mouse line with a Stella-Cre line, but we failed to retrieve any live Mll-AF4-expressing embryos from as early as E8.5 (data not shown). Embryonic lethality following germline expression of Mll-AF4 in this conditional mouse line had been demonstrated previously (Metzler et al., 2006).

Other possible explanations for the lack of a patient-like phenotype in the mouse models include the requirement of a second hit; however, recent sequencing data argue against this, and even KRAS mutations, which are occasionally present, seem to impact on the timing of disease onset and the migration of MLL-AF4-transduced cells, but they are not required for disease initiation (Andersson et al., 2015; Bardini et al., 2011; Prieto et al., 2016; Tamai et al., 2011). Another explanation may be that there are fundamental differences between mouse and human hematopoietic development that prevent progression to B-ALL in the murine context. To address this, we plan to analyze the impact of MLL-AF4 expression in human FL cells in the future. There is good evidence to suggest that the reciprocal fusion gene, AF4-MLL, which is expressed in 50%–80% of patients, plays an integral part in the development of the disease (Bursen et al., 2010; Wilkinson et al., 2013). We tried to co-express AF4-MLL in Mll-AF4-expressing cells by viral transduction; however, due to the very large size of the AF4-MLL construct, transduction efficiencies were extremely low and precluded any further analyses (data not shown). Answering this question may have to await the generation of a true translocator mouse model of the disease.

Finally, it has been reported that BM stromal cells from MLL-AF4+ infant patients express the fusion gene, suggesting a stromal involvement, and that a common progenitor for the

hematopoietic and mesenchymal lineage was the initial target for the translocation (Menendez et al., 2009). We detected low-fusion-transcript expression in BM stromal cells following VEC-Cre-mediated induction. Whether these are the same relevant stromal cell types as in the patients is still unclear. We have expressed MLL-AF4 in stromal cell lines, but we found no significant impact on their hematopoietic supportive activity (data not shown). Culture homeostasis of patient-derived mesenchymal stromal cells also was shown to be normal (Menendez et al., 2009); thus, the role of the microenvironment in MLL-AF4+ infant leukemia requires further investigation. To test this in an in vivo model would require the identification of a common hematopoietic-mesenchymal progenitor and a way of genetically targeting it, an approach we will attempt to take in the future.

In summary, our data demonstrate functional changes in hematopoietic development induced by the Mll-AF4 fusion in the embryo, and they provide a description of a pre-leukemic state. Further molecular dissection of this state may point to hurdles that have to be overcome to achieve progression to pro-B ALL, further informing the biology of this aggressive and often fatal leukemia.

EXPERIMENTAL PROCEDURES

Mice

Heterozygous *Mll-AF4loxP* knockin (Metzler et al., 2006), heterozygous *VEC-Cre* transgenic (Chen et al., 2009), and heterozygous *Vav-Cre* transgenic (Stadtfeld and Graf, 2005) mice were crossed to produce embryos and adult mice of the desired genotypes. For embryo generation, the morning of plug detection was considered day 0. All animals were housed according to institutional guidelines and experimental procedures had UK Home Office approval.

Single-Cell Preparations

Tissues were dissected from embryos (only the region around the chorionic plate of the placenta was used) and dissociated by collagenase treatment (Alfa Aesar; 0.125% in PBS; for AGM, yolk sac, and placenta) or by drawing through a needle attached to a syringe (FL). Adult BM was collected by flushing the femur and tibia, and spleen and liver were mechanically dissociated on top of a filter. Red blood cell lysis (Pharm LyseTM Buffer, Becton Dickinson [BD]) was performed on E14 FL cells, BM, spleen, liver, and peripheral blood. For BM stroma collection, bones were crushed and treated with collagenase.

Flow Cytometry and Cell Sorting

The following antibodies were used to analyze cell populations from tissues or colonies using combinations indicated in the text and/or the figure legends: CD3e-APC (BD I45-2C11), Ter119-APC (eBioscience TER119), F4/80-APC (BioLegend BM8), Nk1.1-APC (BD PK136), Gr1-APC (BD RB6-8C5), IL7R-PE (eBioscience A7T34), Sca1-PB (BioLegend E13-161.7), ckit-APCeF780 (eBioscience 2B8), CD45-AF700 or CD45-FITC (eBioscience 30-F11), Flt3-biotin (eBioscience A2F10), B220-PECy7 (BioLegend RA3-6B2), CD19-PECy7 (BioLegend 1D2) and Qdot655 (Life Technologies), CD24-BV421 (BioLegend M1/69), B220-AF700 (eBioscience RA3-6B2), AA4.1-PECy7 (BioLegend AA4.1), CD19-BV605 (BioLegend 6D5), CD43-FITC (BioLegend 1B11), CD48-APC (eBioscience HM48-1), EPCR-PE (eBioscience 1560), B220-FITC (eBioscience HIS24), CD41-PE (eBioscience MWReg30), CD11b-APC (eBioscience M1/70), Gr1-PE (eBioscience RB6-8C5), CD19-eFluor450 (eBioscience 1D3), CD34-FITC (eBioscience RAM34), CD150-eFluor450 (eBioscience mShad150), CD31-PECy7 (BioLegend 390), and CD51-PE (BioLegend RMV-7). Biotin antibodies were conjugated to Qdot655 streptavidin conjugate (Thermo Fisher Scientific). The lineage cocktail (Lin-) consisted of antibodies against CD3e, Nk1.1, Gr1, Ter119, and F4/80. Dead cells were excluded using SYTOX Dead Cell Stains (Life Technologies) or 7AAD (Invitrogen). Flow cytometry was performed on a CyAn ADP analyzer (Beckman Coulter) or an LSR Fortessa (BD), and data were analyzed using FlowJo software (Tree Star). Cells were sorted using a high-speed Influx or ARIA cell sorter (BD).

Colony-Forming Assays

For myeloid assays, cells were plated in M3434 Methocult (STEMCELL Technologies) in triplicate at different concentrations, and colonies were scored and counted after 7 days. For lymphoid assays, cells were plated in M3630 Methocult (STEMCELL Technologies) supplemented with 20 ng/ml stem cell factor (SCF) and 20 ng/ml Flt3 (PeproTech) in triplicate at different concentrations, and colonies were counted after 14 days. Where stated, DOT1L inhibitors EPZ-5676 and SGC0946 (both Cambridge Bioscience) and BET protein inhibitor I-BET151 (Sigma) were added to the lymphoid methylcellulose medium at the indicated concentrations.

Repopulation Assays

Cell preparations were injected intravenously into irradiated (9.5 Gy) recipients that differed in their CD45 isoform expression. For the primary transplants, E11 AGM, E12 Plac, E12 FL, and E14 FL were injected at 1, 0.5, 0.5, and 0.004 embryo equivalent per recipient, respectively, together with 100,000 total BM helper cells. For the secondary and tertiary transplants, between two and three million total BM cells (without helper cells) were injected, which were normalized according to the level of donor chimerism. At the indicated time points, donor contribution to the peripheral blood was analyzed by flow cytometry using CD45.1-PE (eBioscience A20), CD45.2-FITC (eBioscience 104), CD11b-eFluor450 (eBioscience M1/70), Gr1-AF700 (BioLegend RB6-8C5), CD19-BV605 (BioLegend 1D2), B220-PECy7 (eBioscience RA3-6B2), CD3-APC (BioLegend I45-2C11), and ckit-APCeFluor780 (eBioscience 2B8). Mice were considered positive for repopulation if the donor contribution to the total peripheral blood was >5%. Peripheral blood counts were performed on an ABC blood counter (Woodley).

Immunohistochemistry and Histology

Cryosections were prepared from paraformaldehyde-fixed embryos and stained with anti-Mll1 (Novus Biologicals and Bethyl Laboratories), anti-CD34-FITC (eBioscience), anti-ckit (R&D Systems), followed by secondary antibodies anti-rabbit AlexaFluor555, anti-goat-AlexaFluor555, and anti-rabbit AlexaFluor647 (Abcam). Sections were mounted with DAPI-containing Vectashield mounting medium (Vector Laboratories), images were obtained on an AxioImager Z2 (Zeiss) fitted with a Hammamatsu Flash 4 V2 camera at room temperature, and images were analyzed with the Zen Blue 2012 software.

Tissues from diseased mice were fixed in formalin and embedded in wax. Sections were prepared and stained with H&E, dehydrated, and mounted in DePeX-mounting medium (Fisher Scientific).

Gene Expression Analysis

RNA extraction from total embryonic tissue or sorted cells was achieved with the QIAGEN miRNeasy mini or micro kit. Reverse transcription was carried out with the iScript cDNA Synthesis Kit (Bio-Rad), and cDNA was quantified using Power SYBR according to the manufacturer's instructions. Primers used were the following: MLL-F, 5′-CACGGACTCCTAGAGCGAAC-3′; MLL-R, 5′-TTCTGTGTTGCCTGGACCTC-3′; Mll-AF4-F, 5′-AGTGGGCATGTAGAGGGATC-3′; Mll-AF4-R, 5′-ATGGCTCAGCTGTACTAGGC-3′; β-actin-F, 5′-TCCTGTCCTCACTGTCCA-3′; and β-actin-R, 5′-GTCCGCCTAGAAGCACTTGC-3′. The qPCR reactions were run on an ABI7900HT.

Genotyping and Inversion Efficiencies

To determine the inversion efficiency, a three-way PCR was carried out in which the two forward primers, one for the non-inverted version of the construct (Mll-NonI) and one for the inverted construct (AIR), shared a common reverse primer (Mll-In10). The sequences are as follows: Mll-NonI, 5′-TCGCCTTCTTGACGAGTTCT-3′; AIR, 5′-CAAGGTTTCTAGCACTTACGAATTAATATGC-3′; and Mll-In10, 5′-ATGATGCCACTGTGCTGTGT-3′. These primers also were used for genotyping, together with primers for the detection of Cre recombinase (CreF, 5′-CTGACTGACGGTGGGAGAAT-3′ and CreR, 5′-CTTGCATGATCTCCGGTATT-3′). In some cases, where PCR reactions

only detected the non-inverted allele, primers were included for the *Myogenin* gene (Myo1, 5′-TTACGTCCATCGTGGACAGC-3′ and Myo2, 5′-TGGGCTGGG TGTTAGTCTTA-3′). PCR reactions were run on a Peltier Thermal Cycler.

Statistical Analysis

Graphs and statistical analysis were done using GraphPad Prism 6. Error bars denote SEM and are included where at least three biological replicates were obtained with three technical replicates each. The p values less than 0.05 were considered significant and were obtained using the non-parametric Mann-Whitney test, the non-parametric Kruskal-Wallis test with Dunn's multiple comparisons, or a one-way ANOVA with Tukey's multiple comparisons test, as indicated in the figure legends. Survival rates were determined with Kaplan-Meier survival plots. Where statistical significance was reached, this is indicated by asterisks.

SUPPLEMENTAL INFORMATION

Supplemental Information includes two figures and one table and can be found with this article online at http://dx.doi.org/10.1016/j.celrep.2016.06.046.

AUTHOR CONTRIBUTIONS

N.A.B. and C.M. helped design the study, carried out the majority of the experiments, analyzed the data, and edited the manuscript. Specifically, N.A.B. contributed the immunohistochemistry data, performed functional assays with whole tissues, and analyzed the sick mice, while C.M. contributed qPCR data, performed functional assays with sorted cells, and carried out repopulation assays. C.K. provided assistance with the mouse experiments, genotyping, and analysis of transplantation results. W.A.B. analyzed expression in stromal cells and G.G. assisted with experiments and editing of the manuscript. S.E.W.J. provided unpublished information and edited the manuscript. B.J.H. assisted with experimental design and data analysis and edited the manuscript. K.O. designed and supervised the study, assisted with the experiments, and wrote the paper.

ACKNOWLEDGMENTS

The authors are very grateful to Professor Terence Rabbitts for providing the Mll-AF4 invertor strain, to Professor Nancy Speck for providing the VEC-Cre strain, and to Professor Thomas Graf for providing the Vav-Cre strain. They also would like to acknowledge the Cambridge Institute for Medical Research flow cytometry team, Dr. Reiner Schulte, Dr. Chiara Cossetti, and Michal Maj, and the Edinburgh MRC Centre for Regenerative Medicine flow cytometry team, Fiona Rossi and Dr Claire Cryer, for sorting services; the Cambridge Institute for Medical Research microscopy team, Matthew Gratian and Mark Bowen, for technical support and advice; and the staff of the animal facility for assistance with animal maintenance and experimentation. We also thank Drs. Charlotta Böiers and Joana Carrelha for advice on LMPP-sorting strategies and Drs. Pablo Menéndez and Alejandra Sanjuan-Pla for helpful discussions. Core facilities at the Cambridge Institute for Medical Research are supported by Strategic Award WT100140 and equipment grant 093026; core facilities at the Edinburgh MRC Centre for Regenerative Medicine are supported by centre grant MR/K017047/1. This work was funded by a Bloodwise Bennett Senior Fellowship (10015 to K.O.), a Wellcome Trust Clinical PhD Studentship (097454/z/11/z to N.A.B.) the Gabrielle's Angel Foundation for Cancer Research (to K.O.), and the Kay Kendall Leukaemia Fund (to K.O.).

Received: January 19, 2016
Revised: April 27, 2016
Accepted: June 9, 2016
Published: July 7, 2016

REFERENCES

Adolfsson, J., Månsson, R., Buza-Vidas, N., Hultquist, A., Liuba, K., Jensen, C.T., Bryder, D., Yang, L., Borge, O.J., Thoren, L.A., et al. (2005). Identification of Flt3+ lympho-myeloid stem cells lacking erythro-megakaryocytic potential a revised road map for adult blood lineage commitment. Cell *121*, 295–306.

Alva, J.A., Zovein, A.C., Monvoisin, A., Murphy, T., Salazar, A., Harvey, N.L., Carmeliet, P., and Iruela-Arispe, M.L. (2006). VE-Cadherin-Cre-recombinase transgenic mouse: a tool for lineage analysis and gene deletion in endothelial cells. Dev. Dyn. *235*, 759–767.

Alvarez-Silva, M., Belo-Diabangouaya, P., Salaün, J., and Dieterlen-Lièvre, F. (2003). Mouse placenta is a major hematopoietic organ. Development *130*, 5437–5444.

Andersson, A.K., Ma, J., Wang, J., Chen, X., Gedman, A.L., Dang, J., Nakitandwe, J., Holmfeldt, L., Parker, M., Easton, J., et al.; St. Jude Children's Research Hospital–Washington University Pediatric Cancer Genome Project (2015). The landscape of somatic mutations in infant MLL-rearranged acute lymphoblastic leukemias. Nat. Genet. *47*, 330–337.

Bardini, M., Galbiati, M., Lettieri, A., Bungaro, S., Gorletta, T.A., Biondi, A., and Cazzaniga, G. (2011). Implementation of array based whole-genome high-resolution technologies confirms the absence of secondary copy-number alterations in MLL-AF4-positive infant ALL patients. Leukemia *25*, 175–178.

Böiers, C., Carrelha, J., Lutteropp, M., Luc, S., Green, J.C., Azzoni, E., Woll, P.S., Mead, A.J., Hultquist, A., Swiers, G., et al. (2013). Lymphomyeloid contribution of an immune-restricted progenitor emerging prior to definitive hematopoietic stem cells. Cell Stem Cell *13*, 535–548.

Bueno, C., Montes, R., Melen, G.J., Ramos-Mejia, V., Real, P.J., Ayllón, V., Sanchez, L., Ligero, G., Gutierrez-Aranda, I., Fernández, A.F., et al. (2012). A human ESC model for MLL-AF4 leukemic fusion gene reveals an impaired early hematopoietic-endothelial specification. Cell Res. *22*, 986–1002.

Bursen, A., Schwabe, K., Rüster, B., Henschler, R., Ruthardt, M., Dingermann, T., and Marschalek, R. (2010). The AF4.MLL fusion protein is capable of inducing ALL in mice without requirement of MLL.AF4. Blood *115*, 3570–3579.

Chen, W., Li, Q., Hudson, W.A., Kumar, A., Kirchhof, N., and Kersey, J.H. (2006). A murine Mll-AF4 knock-in model results in lymphoid and myeloid deregulation and hematologic malignancy. Blood *108*, 669–677.

Chen, M.J., Yokomizo, T., Zeigler, B.M., Dzierzak, E., and Speck, N.A. (2009). Runx1 is required for the endothelial to haematopoietic cell transition but not thereafter. Nature *457*, 887–891.

Daigle, S.R., Olhava, E.J., Therkelsen, C.A., Majer, C.R., Sneeringer, C.J., Song, J., Johnston, L.D., Scott, M.P., Smith, J.J., Xiao, Y., et al. (2011). Selective killing of mixed lineage leukemia cells by a potent small-molecule DOT1L inhibitor. Cancer Cell *20*, 53–65.

Daigle, S.R., Olhava, E.J., Therkelsen, C.A., Basavapathruni, A., Jin, L., Boriack-Sjodin, P.A., Allain, C.J., Klaus, C.R., Raimondi, A., Scott, M.P., et al. (2013). Potent inhibition of DOT1L as treatment of MLL-fusion leukemia. Blood *122*, 1017–1025.

Daser, A., and Rabbitts, T.H. (2005). The versatile mixed lineage leukaemia gene MLL and its many associations in leukaemogenesis. Semin. Cancer Biol. *15*, 175–188.

Dawson, M.A., Prinjha, R.K., Dittmann, A., Giotopoulos, G., Bantscheff, M., Chan, W.I., Robson, S.C., Chung, C.W., Hopf, C., Savitski, M.M., et al. (2011). Inhibition of BET recruitment to chromatin as an effective treatment for MLL-fusion leukaemia. Nature *478*, 529–533.

Ernst, P., Fisher, J.K., Avery, W., Wade, S., Foy, D., and Korsmeyer, S.J. (2004). Definitive hematopoiesis requires the mixed-lineage leukemia gene. Dev. Cell *6*, 437–443.

Frame, J.M., McGrath, K.E., and Palis, J. (2013). Erythro-myeloid progenitors: "definitive" hematopoiesis in the conceptus prior to the emergence of hematopoietic stem cells. Blood Cells Mol. Dis. *51*, 220–225.

Gekas, C., Dieterlen-Lièvre, F., Orkin, S.H., and Mikkola, H.K. (2005). The placenta is a niche for hematopoietic stem cells. Dev. Cell *8*, 365–375.

Godin, I., Garcia-Porrero, J.A., Dieterlen-Lièvre, F., and Cumano, A. (1999). Stem cell emergence and hemopoietic activity are incompatible in mouse intraembryonic sites. J. Exp. Med. *190*, 43–52.

Greaves, M. (2005). In utero origins of childhood leukaemia. Early Hum. Dev. *81*, 123–129.

Kent, D.G., Copley, M.R., Benz, C., Wöhrer, S., Dykstra, B.J., Ma, E., Cheyne, J., Zhao, Y., Bowie, M.B., Zhao, Y., et al. (2009). Prospective isolation and molecular characterization of hematopoietic stem cells with durable self-renewal potential. Blood *113*, 6342–6350.

Krause, D.S., and Scadden, D.T. (2015). A hostel for the hostile: the bone marrow niche in hematologic neoplasms. Haematologica *100*, 1376–1387.

Krivtsov, A.V., and Armstrong, S.A. (2007). MLL translocations, histone modifications and leukaemia stem-cell development. Nat. Rev. Cancer *7*, 823–833.

Krivtsov, A.V., Feng, Z., Lemieux, M.E., Faber, J., Vempati, S., Sinha, A.U., Xia, X., Jesneck, J., Bracken, A.P., Silverman, L.B., et al. (2008). H3K79 methylation profiles define murine and human MLL-AF4 leukemias. Cancer Cell *14*, 355–368.

Kumaravelu, P., Hook, L., Morrison, A.M., Ure, J., Zhao, S., Zuyev, S., Ansell, J., and Medvinsky, A. (2002). Quantitative developmental anatomy of definitive haematopoietic stem cells/long-term repopulating units (HSC/RUs): role of the aorta-gonad-mesonephros (AGM) region and the yolk sac in colonisation of the mouse embryonic liver. Development *129*, 4891–4899.

Malouf, C., and Ottersbach, K. (2013). The unconventional embryo: immune-restricted potential precedes multipotentiality. Cell Stem Cell *13*, 509–510.

McGrath, K.E., Frame, J.M., Fegan, K.H., Bowen, J.R., Conway, S.J., Catherman, S.C., Kingsley, P.D., Koniski, A.D., and Palis, J. (2015). Distinct sources of hematopoietic progenitors emerge before HSCs and provide functional blood cells in the mammalian embryo. Cell Rep. *11*, 1892–1904.

Medvinsky, A., and Dzierzak, E. (1996). Definitive hematopoiesis is autonomously initiated by the AGM region. Cell *86*, 897–906.

Medvinsky, A., Rybtsov, S., and Taoudi, S. (2011). Embryonic origin of the adult hematopoietic system: advances and questions. Development *138*, 1017–1031.

Menendez, P., Catalina, P., Rodríguez, R., Melen, G.J., Bueno, C., Arriero, M., García-Sánchez, F., Lassaletta, A., García-Sanz, R., and García-Castro, J. (2009). Bone marrow mesenchymal stem cells from infants with MLL-AF4+ acute leukemia harbor and express the MLL-AF4 fusion gene. J. Exp. Med. *206*, 3131–3141.

Metzler, M., Forster, A., Pannell, R., Arends, M.J., Daser, A., Lobato, M.N., and Rabbitts, T.H. (2006). A conditional model of MLL-AF4 B-cell tumourigenesis using invertor technology. Oncogene *25*, 3093–3103.

Mirshekar-Syahkal, B., Fitch, S.R., and Ottersbach, K. (2014). Concise review: from greenhouse to garden: the changing soil of the hematopoietic stem cell microenvironment during development. Stem Cells *32*, 1691–1700.

Montecino-Rodriguez, E., Li, K., Fice, M., and Dorshkind, K. (2014). Murine B-1 B cell progenitors initiate B-acute lymphoblastic leukemia with features of high-risk disease. J. Immunol. *192*, 5171–5178.

Montes, R., Ayllón, V., Gutierrez-Aranda, I., Prat, I., Hernández-Lamas, M.C., Ponce, L., Bresolin, S., Te Kronnie, G., Greaves, M., Bueno, C., and Menendez, P. (2011). Enforced expression of MLL-AF4 fusion in cord blood CD34+ cells enhances the hematopoietic repopulating cell function and clonogenic potential but is not sufficient to initiate leukemia. Blood *117*, 4746–4758.

Müller, A.M., Medvinsky, A., Strouboulis, J., Grosveld, F., and Dzierzak, E. (1994). Development of hematopoietic stem cell activity in the mouse embryo. Immunity *1*, 291–301.

Ottersbach, K., and Dzierzak, E. (2005). The murine placenta contains hematopoietic stem cells within the vascular labyrinth region. Dev. Cell *8*, 377–387.

Prieto, C., Stam, R.W., Agraz-Doblas, A., Ballerini, P., Camos, M., Castaño, J., Marschalek, R., Bursen, A., Varela, I., Bueno, C., and Menéndez, P. (2016). Activated KRAS cooperates with MLL-AF4 to promote extramedullary engraftment and migration of cord blood CD34+ HSPC but is insufficient to initiate leukemia. Cancer Res. *76*, 2478–2489.

Sanjuan-Pla, A., Bueno, C., Prieto, C., Acha, P., Stam, R.W., Marschalek, R., and Menéndez, P. (2015). Revisiting the biology of infant t(4;11)/MLL-AF4+ B-cell acute lymphoblastic leukemia. Blood *126*, 2676–2685.

Schepers, K., Pietras, E.M., Reynaud, D., Flach, J., Binnewies, M., Garg, T., Wagers, A.J., Hsiao, E.C., and Passegué, E. (2013). Myeloproliferative neoplasia remodels the endosteal bone marrow niche into a self-reinforcing leukemic niche. Cell Stem Cell *13*, 285–299.

Slany, R.K. (2009). The molecular biology of mixed lineage leukemia. Haematologica *94*, 984–993.

Stadtfeld, M., and Graf, T. (2005). Assessing the role of hematopoietic plasticity for endothelial and hepatocyte development by non-invasive lineage tracing. Development *132*, 203–213.

Tamai, H., Miyake, K., Takatori, M., Miyake, N., Yamaguchi, H., Dan, K., Shimada, T., and Inokuchi, K. (2011). Activated K-Ras protein accelerates human MLL/AF4-induced leukemo-lymphomogenicity in a transgenic mouse model. Leukemia *25*, 888–891.

Wilkinson, A.C., Ballabio, E., Geng, H., North, P., Tapia, M., Kerry, J., Biswas, D., Roeder, R.G., Allis, C.D., Melnick, A., et al. (2013). RUNX1 is a key target in t(4;11) leukemias that contributes to gene activation through an AF4-MLL complex interaction. Cell Rep. *3*, 116–127.

Yu, W., Chory, E.J., Wernimont, A.K., Tempel, W., Scopton, A., Federation, A., Marineau, J.J., Qi, J., Barsyte-Lovejoy, D., Yi, J., et al. (2012). Catalytic site remodelling of the DOT1L methyltransferase by selective inhibitors. Nat. Commun. *3*, 1288.

Cell Reports, Volume *16*

Supplemental Information

Mll-AF4 Confers Enhanced Self-Renewal and Lymphoid Potential during a Restricted Window in Development

Neil A. Barrett, Camille Malouf, Chrysa Kapeni, Wendi A. Bacon, George Giotopoulos, Sten Eirik W. Jacobsen, Brian J. Huntly, and Katrin Ottersbach

Supplemental Figures:

Figure S1: Mll-AF4 does not cause major changes to embryonic hematopoiesis. Related to Figure 2.

The proportion of distinct hematopoietic cell populations was determined in embryonic tissues of indicated genotypes by flow cytometry. (A) HSPCs in the E11 YS, n=3-10; (B) HSPCs, n=3-6, and (C) B cells in the E11 AGM, n=3-4; (D) HSPCs, n=3-10; (E) B cells, n=3-10 and (F) LMPPs (Lin- B220- CD19- ckit+ CD45+ Sca1+ Flt3+) in the E12 FL, n=2-7; (G) ESLAM (EPCR+ CD45+ CD150+ CD48-), n=5-8; (H) B cells, n=3-9, and (I) LMPPs in the E14 FL, n=3-7. Error bars = SEM. Statistical analysis was performed using the non-parametric Kruskal-Wallis test with Dunn's multiple comparisons, but no significant differences were found.

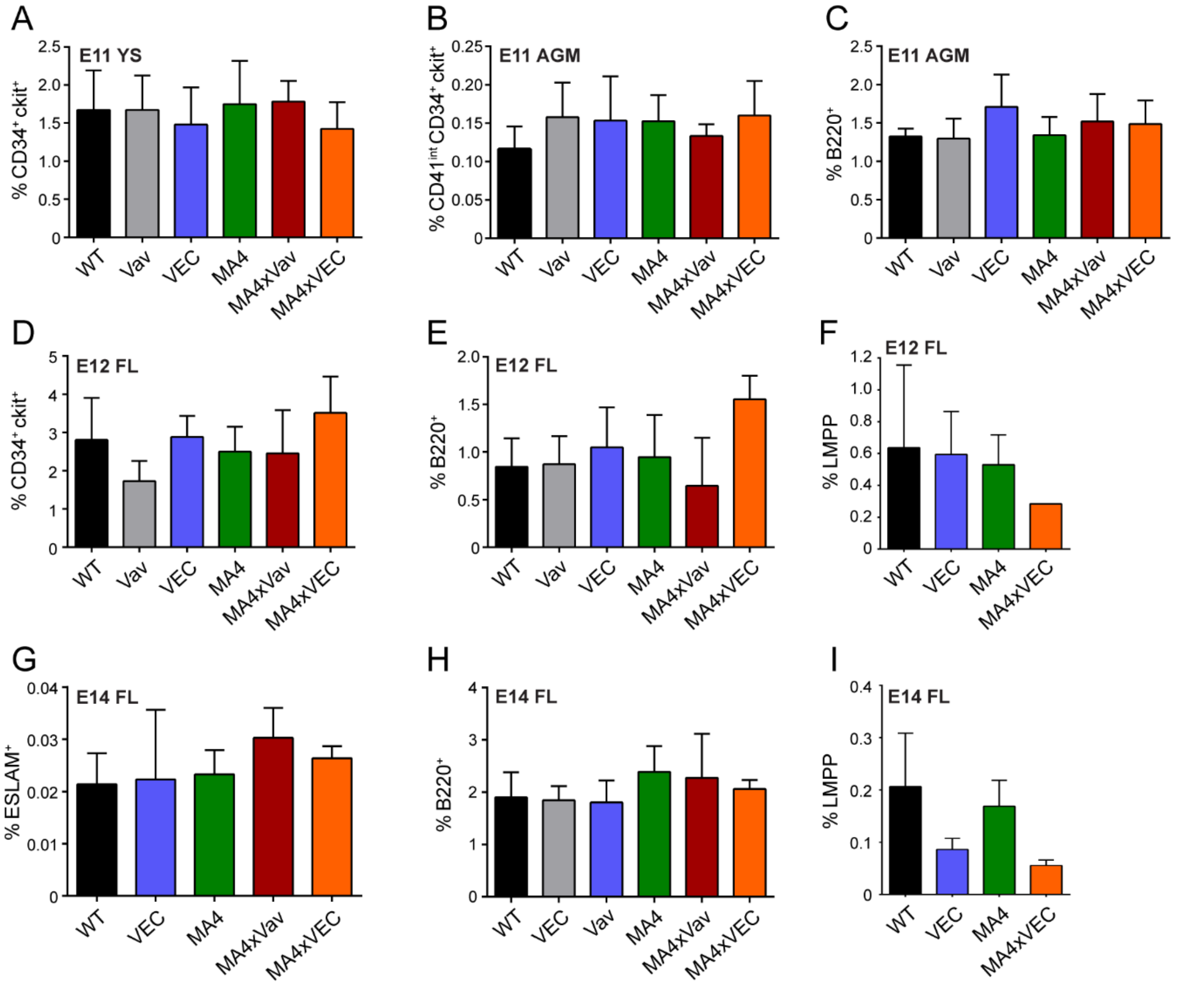

Figure S2: Mll-AF4 recruits SEC and DOT1L. Related to Figure 4.

Effect of inhibitors of BET proteins (IBET) and DOT1L (SGC, EPZ) at indicated concentrations (μM) on B lymphoid colony formation from (A) E14 FL Mll-AF4-expressing cells, n=3-4; (B) E14 FL Cre-only control cells, n=2; (C) adult BM (~1 year old, pre-morbid) Mll-AF4-expressing cells, n=2; (D) adult BM (~1 year old, pre-morbid) Cre-only control cells, n=2. Error bars = SEM. Statistical analysis was performed using a parametric one-way ANOVA with Tukey's multiple comparisons test. **** $p<0.0001$, *** $p<0.001$, ** $p<0.01$.

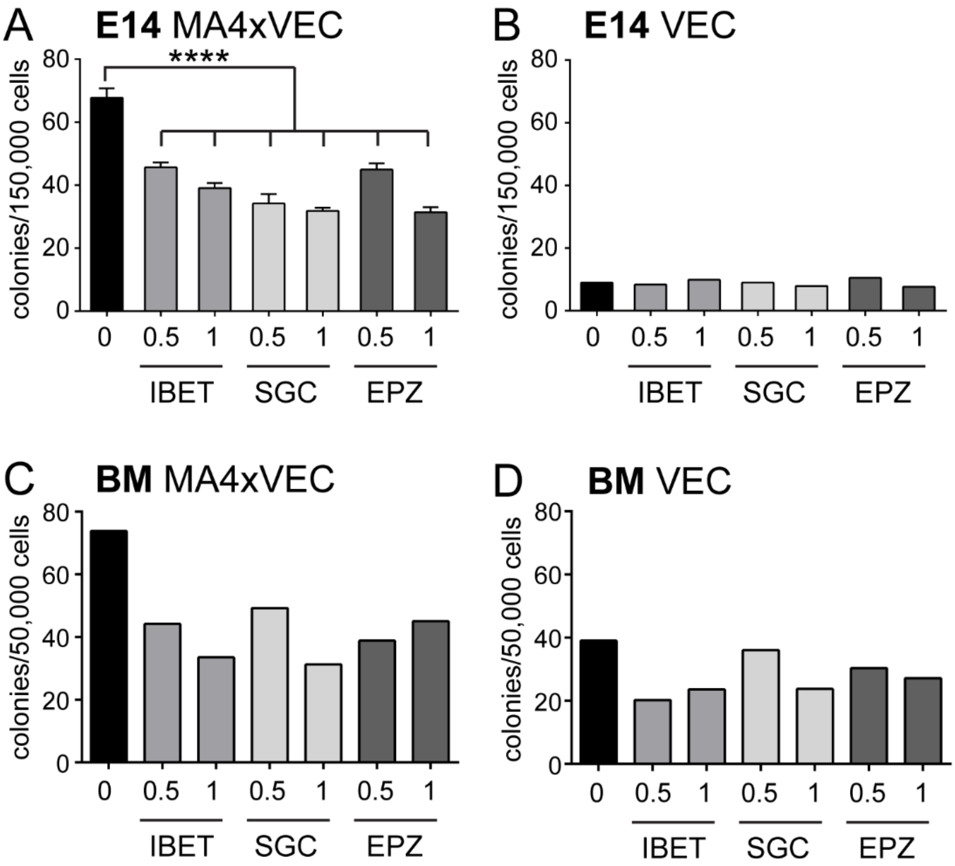

Supplemental Table S1: Disease development in transplant recipients. Related to Figure 5.

	MA4xVEC				VEC		
	No.sick/ total	latency (days)	% donor chimerism	phenotype	No. sick/total	% donor chimerism	phenotype
Primary							
E12 Plac	1/6	186	0	no donor cells<br>expansion of recipient T cells	0/6		
E12 FL	5/7	293-405	95-100	splenomegaly<br>hepatomegaly<br>mesenteric tumor<br>mediastinal disease<br>reduced B cells<br>increased myeloid cells	0/5		
E14 FL	1/14	345	95-100	slight splenomegaly<br>reduced B cells<br>increased myeloid cells<br>increased T cells<br>increased ckit+ cells	1/10	6	expansion of recipient myeloid cells
	2/14	150-186	0-10	slight hepatomegaly<br>expansion of recipient T cells			
Secondary							
E14 FL	1/10	280	95-100	splenomegaly<br>increased myeloid cells<br>increased T cells	1/11	0	splenomegaly<br>slight hepatomegaly<br>reduced recipient leukocytes
